# Supplementary material for: Molecular Dynamics Ensemble Refinement of Intrinsically Disordered Peptides According to Deconvoluted Spectra from Circular Dichroism
Source: Biophys J. 2020 Feb 25;118(7):1665–78. doi: 10.1016/j.bpj.2020.02.015 (PMC7136346; doi:10.1016/j.bpj.2020.02.015)
Supplement: Document S1. Supporting Materials and Methods, Figs. S1–S8, and Tables S1–S11 [file mmc1.pdf]

Biophysical Journal, Volume *118*

Supplemental Information

Molecular Dynamics Ensemble Refinement of Intrinsically Disordered Peptides According to Deconvoluted Spectra from Circular Dichroism

Jacob C. Ezerski, Pengzhi Zhang, Nathaniel C. Jennings, M. Neal Waxham, and Margaret S. Cheung

SUPPLEMENTARY INFORMATION

Tables:

Table S1 The fractions of secondary structures from CAPITO deconvolution of the CD spectra for the CaMKII peptides. Normalized root mean squared deviation (NRMSD) is a dimensionless parameter to assess the goodness of the fitting as defined by Wiedemann et al. [1]

	helix	β-sheet	irregular	NRMSD
RRK	0.32	0.01	0.68	1.55
RAK	0.21	0.01	0.78	0.73
AAA	0.53	0.06	0.41	0.89

Table S2 Fractional secondary structures derived from CD deconvolution using the non-negative least squares fitting method in conjunction with the SDP48, SP37A, SMP56 and SP43 reference data sets. SDP48 is the data set including denatured proteins. Others include the data set of globular proteins.

Data set	Peptide	Helix	Strand	Turn	Unordered	RMSD (Δϵ)
SDP48	RRK	0.04	0.15	0.09	0.72	0.26
	RAK	0.04	0.21	0.13	0.62	0.23
	AAA	0.04	0.34	0.17	0.46	0.36
SP37A	RRK	0.11	0.35	0.20	0.34	2.14
	RAK	0.11	0.35	0.20	0.34	0.97
	AAA	0.08	0.39	0.22	0.32	0.65
SMP56	RRK	0.11	0.35	0.20	0.34	2.14
	RAK	0.11	0.35	0.20	0.34	0.97
	AAA	0.09	0.39	0.20	0.32	0.64
SP43	RRK	0.11	0.35	0.20	0.34	2.14
	RAK	0.11	0.35	0.20	0.34	0.97
	AAA	0.09	0.39	0.20	0.32	0.64

Table S3 Comparison of the structure ensembles of RRK, RAK, and AAA peptides. Root mean square deviation (RMSD) from the averaged structure is calculated for each peptide ensemble based on backbone heavy atoms. The average ($\overline{RMSD}$), standard deviation (σ_{RMSD}), the minimum ($RMSD_{min}$), and maximum ($RMSD_{max}$) values of the RMSD are provided.

	$\overline{RMSD}$ (Å)	σ_{RMSD} (Å)	$RMSD_{min}$ (Å)	$RMSD_{max}$ (Å)	number of structures
RRK	4.6	1.4	2.3	12.5	11002
RAK	4.3	1.7	2.1	12.4	2410
AAA	4.7	1.0	3.5	10.5	130

Table S4 Sequence analysis of RRK, RAK and AAA using CIDER.

peptide	κ	FCR	NCPR	hydropathy	disorder promoting
RRK	0.55	0.25	0.25	4.26	0.6
RAK	0.383	0.2	0.2	4.575	0.6
AAA	0.313	0.1	0.1	5.175	0.6

κ represents an order parameter indicating charge segregation within the peptide; FCR is the fraction of charged residues; NCPR is the linear net charge per residue; hydropathy indicates hydrophobicity and ranges from 0 to 9; the fraction of disorder-promoting residues defined by Dunker [2] is provided.

Table S5 Kullback-Leibler divergence between distributions of total potential energy of the peptides over accumulated simulation time.

Peptide	RRK			RAK			AAA		
Temp (K)	277	285	293	277	285	293	277	285	293
1.2 μs	0.10	0.06	0.02	0.03	0.02	0.05	0.08	0.02	0.02
1.8 μs	0.10	0.01	0.01	0.10	0.02	0.01	0.07	0.01	0.01
2.4 μs	0.04	0.01	0.01	0.06	0.01	0.01	0.04	0.01	0.01
3.0 μs	0.01	N/A		0.03	N/A		0.02	N/A	
3.6 μs	0.01			0.01			0.02		
4.2 μs	0.01			0.01			0.02		
4.8 μs	0.01			0.01			0.01		

Table S6 Details pertaining to the Hieragglo clusters produced by CPPTRAJ are shown for RRK, RAK, and AAA.

peptide	# Cluster	# of frames	fraction	AvgDist (A)	Stdev (A)	Centroid	AvgCDist (A)
RRK	0	7914	0.719	4.5	1.2	9925	8.2
	1	2292	0.208	4.0	1.3	4519	7.7
	2	171	0.016	4.8	1.3	5228	7.1
	3	148	0.013	4.6	1.3	1796	7.0
	4	135	0.012	5.2	1.2	2519	6.9
	5	105	0.01	4.2	1.7	90	7.5
	6	98	0.009	4.7	0.9	16	8.3
	7	87	0.008	5.2	1.0	450	6.9
	8	47	0.004	4.8	1.2	2366	7.4
	9	5	0.0	4.3	1.3	10996	7.2
RAK	0	1883	0.781	4.5	1.6	2042	7.7
	1	197	0.082	4.9	1.2	284	7.1
	2	109	0.045	4.7	1.1	186	8.3
	3	104	0.043	4.4	1.8	1047	8.1
	4	62	0.026	4.1	1.5	1421	7.2
	5	25	0.01	4.1	1.4	608	7.0
	6	14	0.006	4.2	1.3	175	7.4
	7	6	0.002	4.5	0.8	166	7.0
	8	5	0.002	4.7	1.3	233	6.9
	9	5	0.002	3.5	1.0	1532	7.3
AAA	0	59	0.454	2.1	0.8	70	6.6
	1	27	0.208	2.5	0.9	19	6.2
	2	25	0.192	2.4	0.9	41	5.5
	3	9	0.069	1.5	0.5	52	6.0
	4	3	0.023	1.1	0.1	28	5.9
	5	3	0.023	1.9	0.3	128	5.5
	6	1	0.008	0.0	0.0	124	6.9
	7	1	0.008	0	0	125	6.5
	8	1	0.008	0	0	126	5.4
	9	1	0.008	0	0	130	6.9

Table S7 Performance indices for varying subsets of unordered content indicated by a minimum structure content cutoff value. The number of proteins satisfying the cutoff criteria is given by column N. Cells highlighted in red indicate CDPro algorithms are the highest performing methods and cells highlighted in green indicate NN-LSQ is the best performing method.

cut(unorder)	N	Method	σ(H)	r(H)	σ(β)	r(β)	σ(T)	r(T)	σ(U)	r(U)	σ	r
0	411	NN-LSQ	0.1223	0.6953	0.1064	0.6254	0.0623	0.3225	0.1064	0.1601	0.1018	0.6006
		SELCON3	0.1156	0.7023	0.0902	0.6455	0.0537	0.294	0.0863	0.2236	0.0892	0.7147
		CDSSTR	0.1196	0.7255	0.0855	0.7117	0.0604	0.2689	0.0963	0.2519	0.0929	0.7173
		CONTIN/LL	0.1073	0.7632	0.0881	0.698	0.0557	0.2396	0.0921	0.244	0.0878	0.7286
0.1	409	NN-LSQ	0.1213	0.6897	0.1062	0.6239	0.0623	0.3189	0.106	0.1505	0.1014	0.5962
		SELCON3	0.1147	0.696	0.0904	0.642	0.0518	0.31	0.0856	0.2205	0.0885	0.7119
		CDSSTR	0.119	0.7207	0.0856	0.7092	0.06	0.2692	0.0963	0.2318	0.0927	0.7122
		CONTIN/LL	0.1064	0.7595	0.0883	0.6952	0.0552	0.2369	0.0919	0.2276	0.0874	0.7243
0.2	382	NN-LSQ	0.1135	0.656	0.1024	0.6206	0.0631	0.2836	0.1047	0.0967	0.0979	0.561
		SELCON3	0.1137	0.599	0.0868	0.6354	0.0507	0.1805	0.0866	0.0808	0.0874	0.6555
		CDSSTR	0.1158	0.6295	0.0826	0.7097	0.0582	0.1305	0.0978	0.0368	0.0911	0.6511
		CONTIN/LL	0.1076	0.6698	0.0881	0.6615	0.0531	0.1002	0.0934	0.0859	0.0879	0.6582
0.3	31	NN-LSQ	0.101	0.8088	0.0949	0.7596	0.0801	-0.0088	0.2653	-0.1158	0.1549	0.5588
		SELCON3	0.1666	0.6069	0.1163	0.6399	0.0565	0.4048	0.2631	-0.2124	0.1686	0.4654
		CDSSTR	0.1686	0.7112	0.0964	0.7513	0.0647	0.3546	0.2836	-0.1656	0.1749	0.4766
		CONTIN/LL	0.1274	0.7705	0.097	0.7864	0.0872	-0.0871	0.2837	-0.2301	0.1686	0.5025
0.4	10	NN-LSQ	0.079	0.8621	0.1032	0.6008	0.1256	-0.6799	0.4481	-0.5362	0.2416	0.4066
		SELCON3	0.2378	0.2569	0.155	0.0611	0.0732	0.2234	0.4434	-0.4839	0.2658	0.203
		CDSSTR	0.2213	0.4954	0.1025	0.5432	0.086	-0.0818	0.4664	-0.4174	0.2667	0.2552
		CONTIN/LL	0.1525	0.6468	0.1197	0.516	0.136	-0.7972	0.4739	-0.5733	0.2649	0.2813
0.5	3	NN-LSQ	0.0938	0.9636	0.0984	-0.9721	0.0536	0.9672	0.7507	0.9204	0.3824	0.4693
		SELCON3	0.159	0.6424	0.0957	-0.7226	0.0593	0.4159	0.7767	0.5898	0.4004	0.236
		CDSSTR	0.2305	0.6337	0.0797	-0.6898	0.0953	0.5752	0.7986	0.6375	0.4202	0.152
		CONTIN/LL	0.1913	0.618	0.0945	-0.7393	0.0615	0.5737	0.7984	0.4635	0.4144	0.1531

Table S8 Performance indices for varying subsets of beta content indicated by a minimum structure content cutoff value. The number of proteins satisfying the cutoff criteria is given by column N. Cells highlighted in red indicate CDPro algorithms are the highest performing methods and cells highlighted in green indicate NN-LSQ is the best performing method.

cut(Strand)	N	Method	σ(H)	r(H)	σ(β)	r(β)	σ(T)	r(T)	σ(U)	r(U)	σ	r
0	411	NN-LSQ	0.1223	0.6953	0.1064	0.6254	0.0623	0.3225	0.1064	0.1601	0.1018	0.6006
		SELCON3	0.1156	0.7023	0.0902	0.6455	0.0537	0.294	0.0863	0.2236	0.0892	0.7147
		CDSSTR	0.1196	0.7255	0.0855	0.7117	0.0604	0.2689	0.0963	0.2519	0.0929	0.7173
		CONTIN/LL	0.1073	0.7632	0.0881	0.698	0.0557	0.2396	0.0921	0.244	0.0878	0.7286
0.1	362	NN-LSQ	0.113	0.6376	0.106	0.5707	0.0626	-0.069	0.1066	0.0882	0.0991	0.5114
		SELCON3	0.1188	0.5111	0.0936	0.5689	0.0467	0.0064	0.0869	0.1162	0.0903	0.5952
		CDSSTR	0.1195	0.5548	0.0889	0.6485	0.0514	-0.0428	0.0948	0.1028	0.0919	0.6067
		CONTIN/LL	0.1095	0.6211	0.0921	0.6312	0.048	-0.0702	0.0925	0.1473	0.0885	0.6195
0.2	58	NN-LSQ	0.0878	0.6399	0.1151	0.6107	0.0571	0.0995	0.0892	0.365	0.0897	0.7253
		SELCON3	0.1462	0.3413	0.1412	0.5324	0.0669	0.0429	0.0938	0.3107	0.1168	0.5429
		CDSSTR	0.1315	0.4752	0.1201	0.5896	0.0557	0.2898	0.0862	0.4486	0.1028	0.6633
		CONTIN/LL	0.1032	0.556	0.1169	0.6527	0.0742	-0.2346	0.1062	0.4373	0.1014	0.6769
0.3	42	NN-LSQ	0.0922	0.4555	0.1288	0.5183	0.0592	0.0527	0.087	0.4293	0.0951	0.7644
		SELCON3	0.1648	-0.0426	0.1584	0.282	0.0578	0.2556	0.0977	0.3222	0.1276	0.5595
		CDSSTR	0.1445	0.0452	0.1285	0.4957	0.0595	0.2715	0.0921	0.4674	0.1112	0.6826
		CONTIN/LL	0.1125	0.0946	0.1265	0.5406	0.079	-0.216	0.1096	0.4625	0.1083	0.7024
0.4	23	NN-LSQ	0.0782	0.4687	0.1519	0.1128	0.0662	-0.3074	0.0916	0.1556	0.1024	0.8132
		SELCON3	0.1897	0.0128	0.2007	0.2033	0.0562	0.4744	0.0973	0.0146	0.149	0.536
		CDSSTR	0.1582	0.0748	0.1526	0.1849	0.0595	0.4935	0.0903	-0.1077	0.1225	0.7038
		CONTIN/LL	0.108	0.0064	0.1489	0.1324	0.0937	-0.3867	0.1228	-0.0335	0.1201	0.7204
0.5	7	NN-LSQ	0.0925	0.0935	0.2384	-0.1961	0.0716	0.6353	0.1223	0.461	0.1462	0.7712
		SELCON3	0.206	0.7873	0.265	0.3653	0.0862	0.4311	0.079	0.1478	0.1777	0.5785
		CDSSTR	0.1785	0.8185	0.232	0.3343	0.0884	0.3004	0.0614	0.1436	0.1559	0.7061
		CONTIN/LL	0.0956	0.883	0.1888	0.5545	0.1081	-0.4405	0.0976	0.3249	0.1285	0.8417

Table S9 Performance indices for varying subsets of helix content indicated by a minimum structure content cutoff value. The number of proteins satisfying the cutoff criteria is given by column N. Cells highlighted in red indicate CDPro algorithms are the best performing methods.

cut(Helix)	N	Method	σ(H)	r(H)	σ(β)	r(β)	σ(T)	r(T)	σ(U)	r(U)	σ	r
0	411	NN-LSQ	0.1223	0.6953	0.1064	0.6254	0.0623	0.3225	0.1064	0.1601	0.1018	0.6006
		SELCON3	0.1156	0.7023	0.0902	0.6455	0.0537	0.294	0.0863	0.2236	0.0892	0.7147
		CDSSTR	0.1196	0.7255	0.0855	0.7117	0.0604	0.2689	0.0963	0.2519	0.0929	0.7173
		CONTIN/LL	0.1073	0.7632	0.0881	0.698	0.0557	0.2396	0.0921	0.244	0.0878	0.7286
0.3	349	NN-LSQ	0.1284	0.5175	0.1051	0.4096	0.06	0.4654	0.1059	0.0661	0.1029	0.5835
		SELCON3	0.1095	0.7455	0.079	0.4808	0.0492	0.3902	0.0851	0.1833	0.0835	0.749
		CDSSTR	0.1178	0.7344	0.0789	0.5047	0.0593	0.3481	0.0965	0.2099	0.0907	0.7298
		CONTIN/LL	0.1094	0.7416	0.0828	0.4907	0.0492	0.4211	0.0874	0.1843	0.085	0.7418
0.4	60	NN-LSQ	0.1754	0.4266	0.1072	0.382	0.0561	0.8437	0.1026	0.2175	0.1183	0.7966
		SELCON3	0.1019	0.8184	0.0626	0.5809	0.0841	0.6933	0.0788	0.3919	0.083	0.9123
		CDSSTR	0.1307	0.7961	0.0582	0.6292	0.0975	0.7272	0.0991	0.4106	0.0997	0.9033
		CONTIN/LL	0.1024	0.7996	0.0592	0.5723	0.0881	0.7568	0.0855	0.465	0.0852	0.9111
0.5	29	NN-LSQ	0.2175	0.0229	0.1313	0.0433	0.0431	0.3811	0.1121	0.1085	0.1405	0.8237
		SELCON3	0.0956	0.6264	0.0473	0.4151	0.0772	-0.1275	0.0753	0.3274	0.0758	0.9557
		CDSSTR	0.1428	0.5189	0.0556	0.3421	0.0786	0.1437	0.0994	0.5186	0.0994	0.9474
		CONTIN/LL	0.0969	0.5642	0.0453	0.4476	0.0676	0.1969	0.0663	0.5269	0.0714	0.9611
0.6	18	NN-LSQ	0.251	0.125	0.1415	0.5355	0.042	0.2448	0.1176	0.213	0.157	0.8304
		SELCON3	0.1089	0.3133	0.0378	0.1845	0.0946	-0.5374	0.0765	0.1061	0.0838	0.957
		CDSSTR	0.1509	0.1306	0.0482	0.2191	0.0864	-0.037	0.0936	0.2677	0.1016	0.9577
		CONTIN/LL	0.1056	0.173	0.0308	0.4261	0.0809	-0.145	0.0579	0.3976	0.0742	0.9663
0.7	8	NN-LSQ	0.2755	0.1155	0.1265	0.3087	0.0348	0.1753	0.1516	0.0521	0.1704	0.8552
		SELCON3	0.1138	-0.2914	0.046	0.2832	0.0531	-0.1667	0.0866	-0.4144	0.0797	0.9649
		CDSSTR	0.1356	-0.3952	0.0506	0.4432	0.0743	-0.6562	0.0718	-0.1813	0.0889	0.9664
		CONTIN/LL	0.1067	-0.4052	0.0361	0.6878	0.0673	-0.2457	0.0688	-0.1383	0.0741	0.9685

Table S10 Performance indices for varying subsets of turn content indicated by a minimum structure content cutoff value. The number of proteins satisfying the cutoff criteria is given by column N. Cells highlighted in red indicate CDPro algorithms are the highest performing methods and cells highlighted in green indicate NN-LSQ is the best performing method.

cut(Turn)	N	Method	σ(H)	r(H)	σ(β)	r(β)	σ(T)	r(T)	σ(U)	r(U)	σ	r
0	411	NN-LSQ	0.1223	0.6953	0.1064	0.6254	0.0623	0.3225	0.1064	0.1601	0.1018	0.6006
		SELCON3	0.1156	0.7023	0.0902	0.6455	0.0537	0.294	0.0863	0.2236	0.0892	0.7147
		CDSSTR	0.1196	0.7255	0.0855	0.7117	0.0604	0.2689	0.0963	0.2519	0.0929	0.7173
		CONTIN/LL	0.1073	0.7632	0.0881	0.698	0.0557	0.2396	0.0921	0.244	0.0878	0.7286
0.1	402	NN-LSQ	0.1232	0.6707	0.1059	0.5997	0.0611	0.3253	0.1067	0.1582	0.1019	0.5795
		SELCON3	0.1099	0.729	0.0859	0.6544	0.0528	0.2288	0.0854	0.2365	0.0859	0.7226
		CDSSTR	0.1157	0.7373	0.083	0.7097	0.0594	0.2168	0.0938	0.2802	0.0903	0.7202
		CONTIN/LL	0.1063	0.7619	0.0869	0.683	0.0523	0.2454	0.0895	0.2758	0.086	0.7271
0.2	207	NN-LSQ	0.1029	0.6945	0.1006	0.6267	0.0796	0.4874	0.1335	0.1467	0.1059	0.4636
		SELCON3	0.0927	0.6837	0.0774	0.7	0.0628	0.0373	0.1121	0.101	0.0882	0.6393
		CDSSTR	0.098	0.6848	0.0724	0.7553	0.0757	0.0866	0.1229	0.0434	0.0945	0.6235
		CONTIN/LL	0.0914	0.7171	0.0805	0.7055	0.0636	-0.0783	0.1203	0.0946	0.0913	0.6309
0.3	20	NN-LSQ	0.0866	0.6613	0.0617	0.8478	0.0912	-0.2969	0.094	0.1072	0.0844	0.736
		SELCON3	0.0829	0.7039	0.0712	0.777	0.1307	-0.7941	0.1053	-0.6129	0.1001	0.6196
		CDSSTR	0.0792	0.7744	0.0714	0.8433	0.1397	-0.1234	0.1146	0.0775	0.1049	0.6258
		CONTIN/LL	0.0819	0.6846	0.0663	0.8479	0.1366	-0.6951	0.1216	0.2223	0.1055	0.5872

Table S11 PCDDB entries for the selected CD spectra. The 411 spectra are for proteins with known PDB entries.

CD0000001000	CD0000055000	CD0001158000	CD0003670000	CD0003992009	CD0003996005	CD0004000001	CD0004003011
CD0000002000	CD0000056000	CD0001159000	CD0003671000	CD0003992010	CD0003996006	CD0004000002	CD0004003012
CD0000003000	CD0000057000	CD0001160000	CD0003672000	CD0003992011	CD0003996007	CD0004000003	CD0004003013
CD0000004000	CD0000058000	CD0001161000	CD0003675000	CD0003992012	CD0003996008	CD0004000004	CD0004004000
CD0000005000	CD0000059000	CD0001162000	CD0003675001	CD0003992013	CD0003996009	CD0004000005	CD0004004001
CD0000006000	CD0000060000	CD0001163000	CD0003675002	CD0003993000	CD0003996010	CD0004000006	CD0004004002
CD0000007000	CD0000061000	CD0001164000	CD0003675003	CD0003993001	CD0003996011	CD0004000007	CD0004004003
CD0000008000	CD0000062000	CD0001165000	CD0003675004	CD0003993002	CD0003996012	CD0004000008	CD0004004004
CD0000009000	CD0000063000	CD0001166000	CD0003675005	CD0003993003	CD0003996013	CD0004000009	CD0004004005
CD0000010000	CD0000064000	CD0001167000	CD0003675006	CD0003993004	CD0003997000	CD0004000010	CD0004004006
CD0000011000	CD0000065000	CD0001168000	CD0003675007	CD0003993005	CD0003997001	CD0004000011	CD0004004007
CD0000012000	CD0000067000	CD0001169000	CD0003675008	CD0003993006	CD0003997002	CD0004000012	CD0004004008
CD0000013000	CD0000068000	CD0001170000	CD0003675009	CD0003993007	CD0003997003	CD0004000013	CD0004004009
CD0000014000	CD0000069000	CD0001171000	CD0003675010	CD0003993008	CD0003997004	CD0004001000	CD0004004010
CD0000015000	CD0000070000	CD0001172000	CD0003675011	CD0003993009	CD0003997005	CD0004001001	CD0004004012
CD0000016000	CD0000071000	CD0001173000	CD0003675012	CD0003993010	CD0003997006	CD0004001002	CD0004004013
CD0000017000	CD0000099000	CD0001174000	CD0003675013	CD0003993011	CD0003997007	CD0004001003	CD0004005000
CD0000018000	CD0000100000	CD0001175000	CD0003690000	CD0003993012	CD0003997008	CD0004001004	CD0004005001
CD0000019000	CD0000101000	CD0001176000	CD0003889000	CD0003993013	CD0003997009	CD0004001005	CD0004005002
CD0000020000	CD0000102000	CD0001177000	CD0003890000	CD0003994000	CD0003997010	CD0004001006	CD0004005003
CD0000021000	CD0000103000	CD0001178000	CD0003891000	CD0003994001	CD0003997011	CD0004001007	CD0004005004
CD0000022000	CD0000104000	CD0001179000	CD0003892000	CD0003994002	CD0003997012	CD0004001008	CD0004005005
CD0000023000	CD0000105000	CD0001180000	CD0003893000	CD0003994003	CD0003997013	CD0004001009	CD0004005006
CD0000024000	CD0000106000	CD0001181000	CD0003894000	CD0003994004	CD0003998000	CD0004001010	CD0004005008
CD0000025000	CD0000107000	CD0001182000	CD0003896000	CD0003994005	CD0003998001	CD0004001011	CD0004005009
CD0000026000	CD0000108000	CD0001183000	CD0003897000	CD0003994006	CD0003998002	CD0004001012	CD0004005010
CD0000027000	CD0000109000	CD0001184000	CD0003898000	CD0003994007	CD0003998003	CD0004001013	CD0004005011
CD0000028000	CD0000110000	CD0001185000	CD0003900000	CD0003994008	CD0003998004	CD0004002000	CD0004005012
CD0000029000	CD0000111000	CD0001186000	CD0003930000	CD0003994009	CD0003998005	CD0004002001	CD0004005013
CD0000030000	CD0000112000	CD0001187000	CD0003991000	CD0003994010	CD0003998006	CD0004002002	CD0004006000
CD0000031000	CD0000113000	CD0001188000	CD0003991001	CD0003994011	CD0003998007	CD0004002003	CD0004006001
CD0000032000	CD0000114000	CD0001189000	CD0003991002	CD0003994012	CD0003998008	CD0004002004	CD0004006002
CD0000034000	CD0000115000	CD0001190000	CD0003991003	CD0003994013	CD0003998009	CD0004002005	CD0004006003
CD0000035000	CD0000116000	CD0001191000	CD0003991004	CD0003995000	CD0003998010	CD0004002006	CD0004006004
CD0000036000	CD0000117000	CD0001192000	CD0003991005	CD0003995001	CD0003998011	CD0004002007	CD0004006005
CD0000037000	CD0000118000	CD0001193000	CD0003991006	CD0003995002	CD0003998012	CD0004002008	CD0004006006
CD0000038000	CD0000119000	CD0001194000	CD0003991007	CD0003995003	CD0003998013	CD0004002009	CD0004006007
CD0000039000	CD0000120000	CD0001195000	CD0003991008	CD0003995004	CD0003999000	CD0004002010	CD0004006008
CD0000040000	CD0000121000	CD0001196000	CD0003991009	CD0003995005	CD0003999001	CD0004002011	CD0004006009
CD0000041000	CD0000122000	CD0001197000	CD0003991010	CD0003995006	CD0003999002	CD0004002012	CD0004006010
CD0000042000	CD0000123000	CD0001198000	CD0003991011	CD0003995007	CD0003999003	CD0004002013	CD0004006011
CD0000043000	CD0000124000	CD0001199000	CD0003991012	CD0003995008	CD0003999004	CD0004003000	CD0004006012
CD0000044000	CD0000125000	CD0001200000	CD0003991013	CD0003995009	CD0003999005	CD0004003001	CD0004006013
CD0000045000	CD0000126000	CD0001201000	CD0003992000	CD0003995010	CD0003999006	CD0004003002	CD0004244000
CD0000047000	CD0000127000	CD0001202000	CD0003992001	CD0003995011	CD0003999007	CD0004003003	CD0004676000
CD0000048000	CD0000128000	CD0001203000	CD0003992002	CD0003995012	CD0003999008	CD0004003004	CD0004677000
CD0000049000	CD0001152000	CD0001204000	CD0003992003	CD0003995013	CD0003999009	CD0004003005	CD0004678000
CD0000050000	CD0001153000	CD0001205000	CD0003992004	CD0003996000	CD0003999010	CD0004003006	
CD0000051000	CD0001154000	CD0001206000	CD0003992005	CD0003996001	CD0003999011	CD0004003007	
CD0000052000	CD0001155000	CD0001207000	CD0003992006	CD0003996002	CD0003999012	CD0004003008	
CD0000053000	CD0001156000	CD0003668000	CD0003992007	CD0003996003	CD0003999013	CD0004003009	
CD0000054000	CD0001157000	CD0003669000	CD0003992008	CD0003996004	CD0004000000	CD0004003010	

Figures:

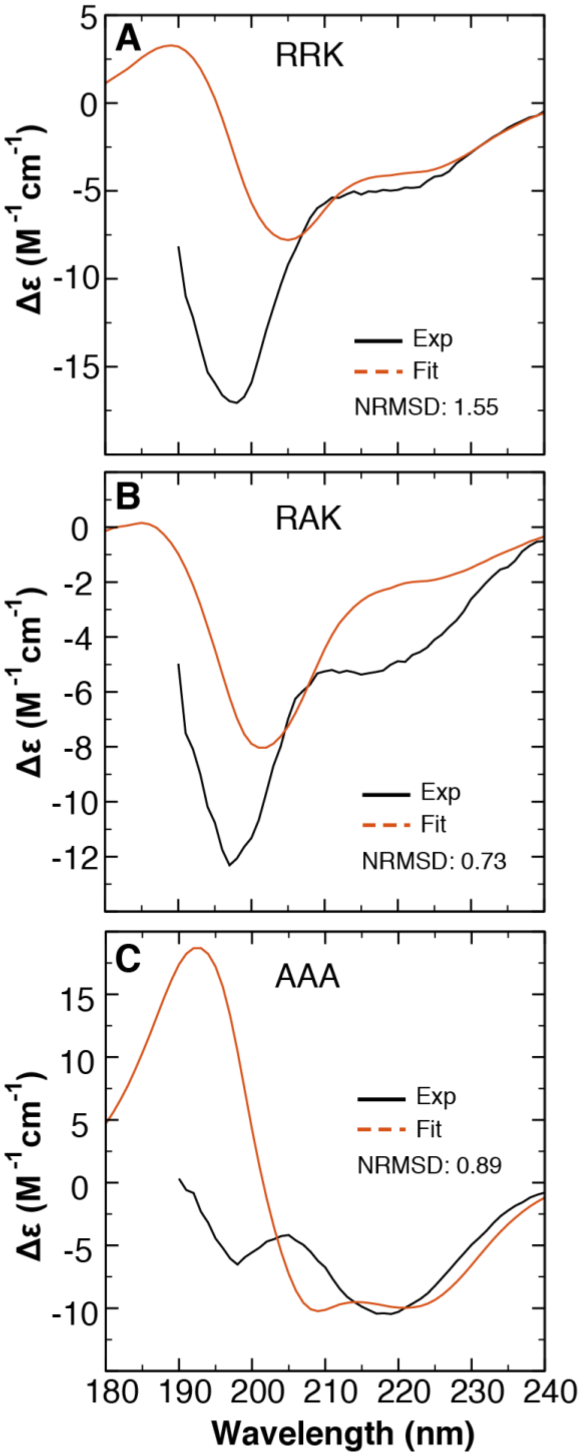

Figure S1 The calculated CD spectra derived from CAPITO for the CaMKII peptides are compared with the experimental data.

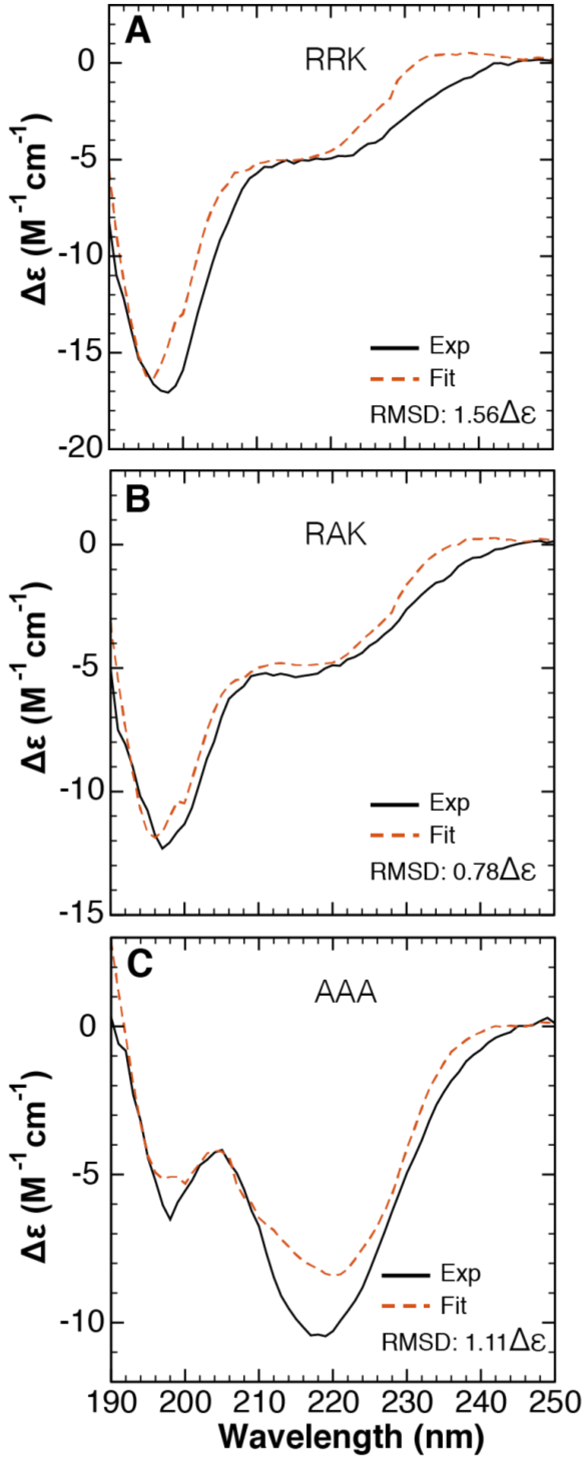

Figure S2 The predicted CD spectra derived from BeStSel for the CaMKII peptides are compared with the experimental data.

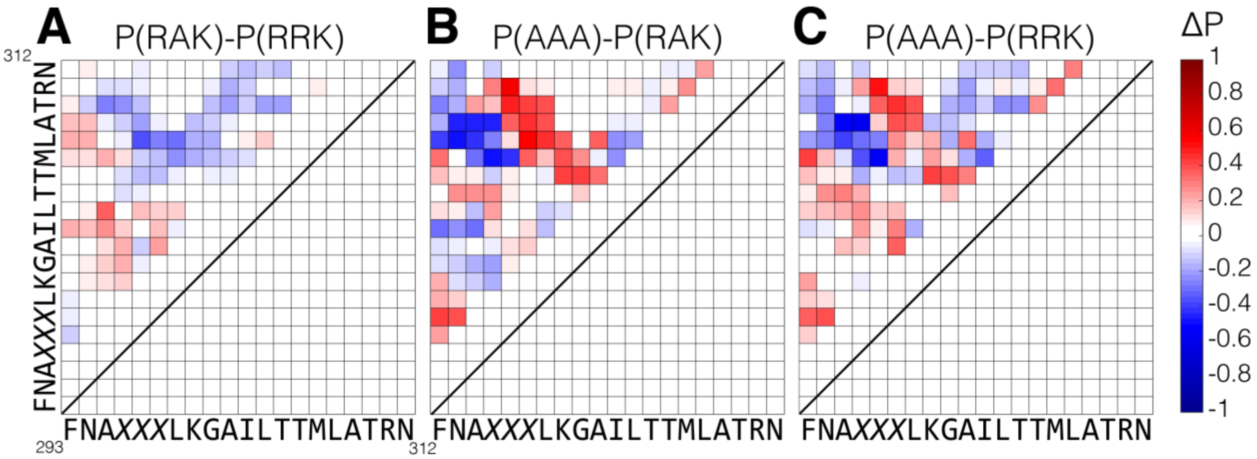

Figure S3 Difference in the probability of contact formation between the CD-refined MD structures of CaMKII peptides. Probability of contact formation are compared between peptide RAK and the wildtype RRK (a), between peptides AAA and RAK (b), and between peptide AAA and the wildtype RRK (c). The amino acid sequences are provided as the axis labels (X refers to any of the three residues RRK/RAK/AAA for corresponding peptides). The criteria of the contact formation can be found in the Method section in the main text.

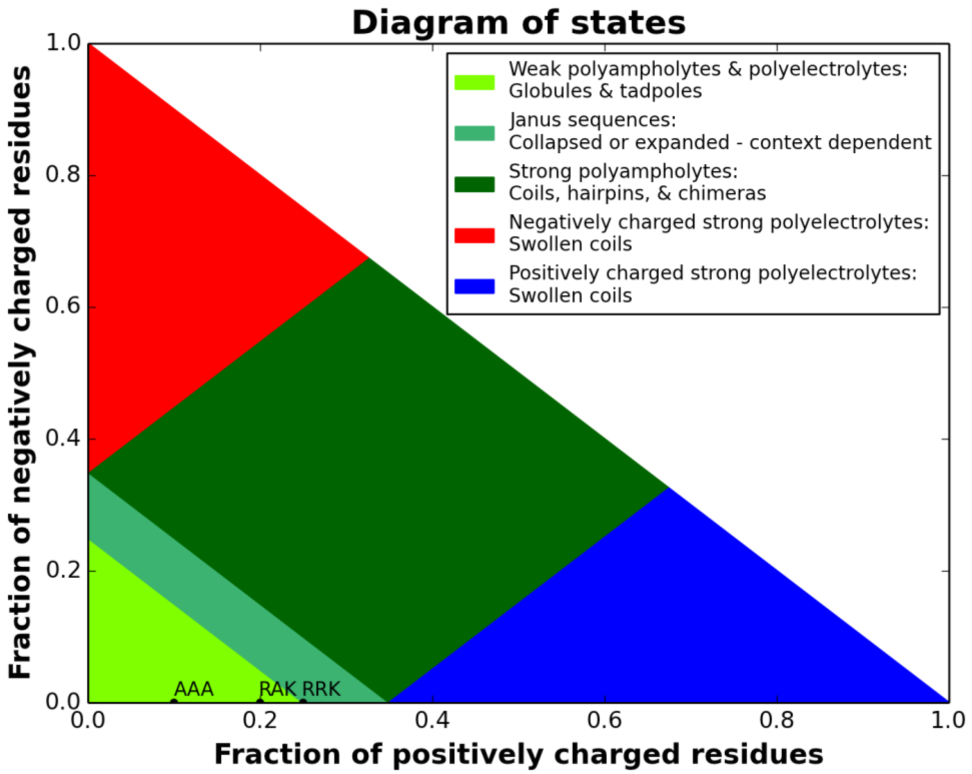

Figure S4 Diagram of states from CIDER analysis for the CaMKII peptides. The distribution of charged residues is indicative of ensemble conformation. RRK is in line with an expanded conformational ensemble, whereas RAK and AAA are predicted to more ordered.

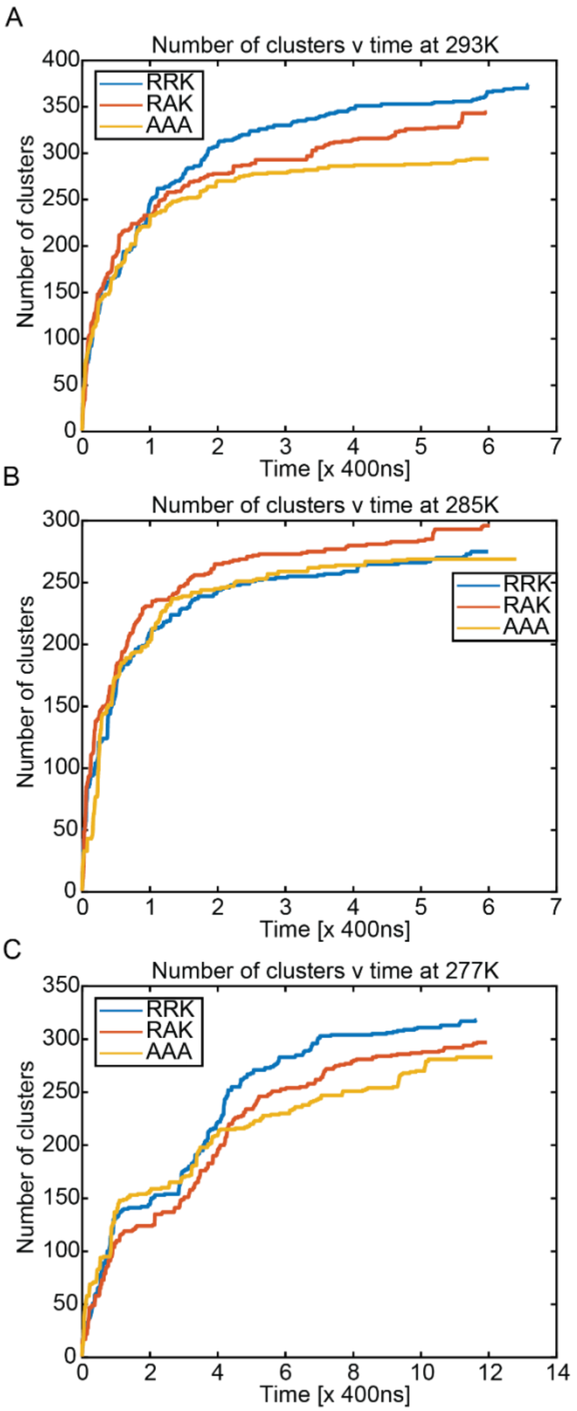

Figure S5 Clustering analysis for the MD trajectories of CaMKII peptides. Number of clusters versus accumulated time were plotted for RRK, RAK and AAA peptides at simulation temperatures of (A) 277K, (B) 285K and (C) 293K. Trajectories belonging to each peptide/temperature combination are concatenated together in chronological order and clustered using an algorithm described by Daura et al. [3]. A cutoff of 2.5 angstroms is used to distinguish clusters by root mean square distance of backbone C_α atoms. Clusters were generated using every 10_{th} frame from the MD trajectories.

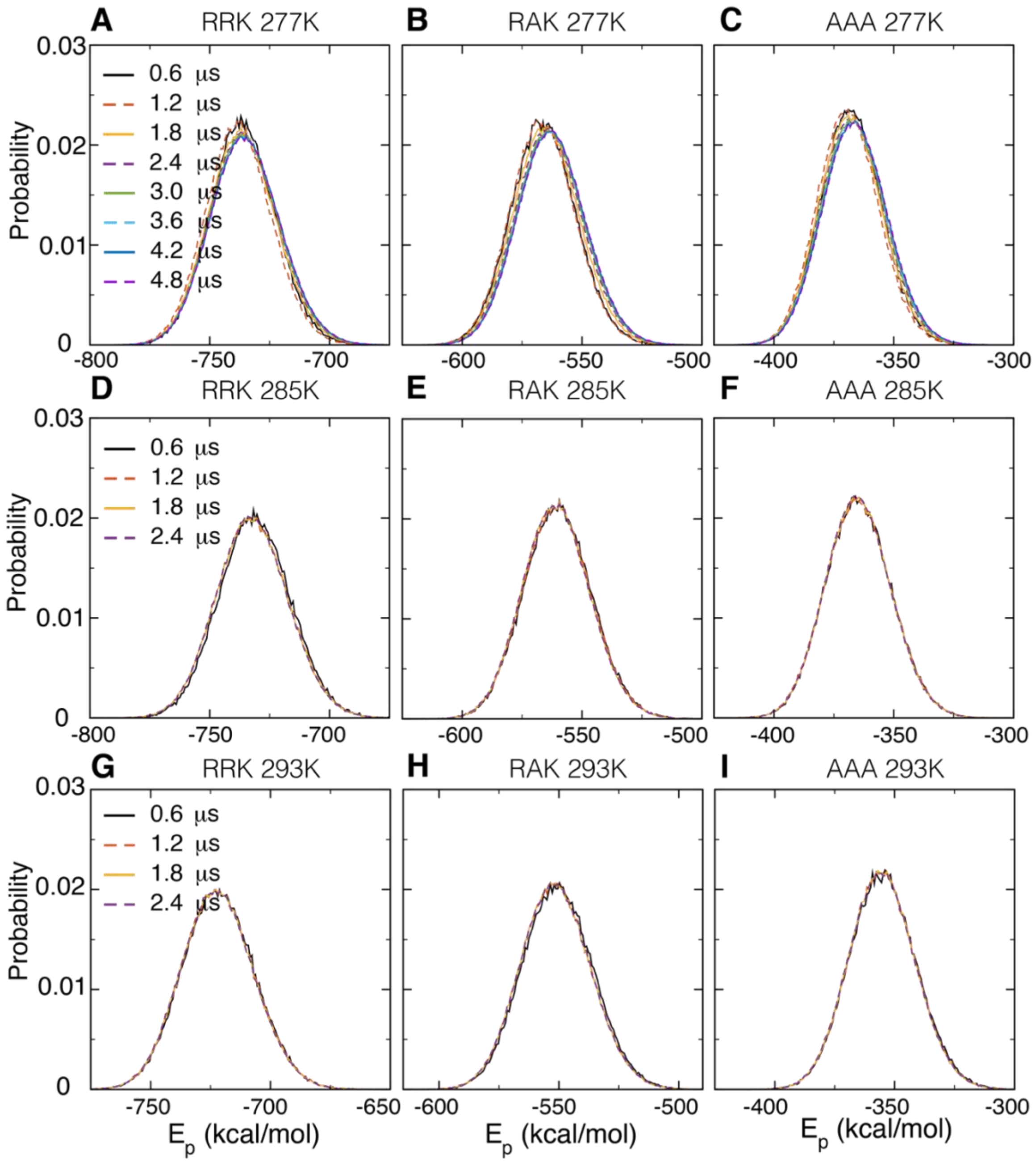

Figure S6 Probability distribution of the potential energy (E_p) for the MD trajectories of CaMKII peptides. The probability distribution of the potential energy for the peptides were compared for each peptide at temperatures of 277K (A, B, C, respectively), 285 K (D, E, F, respectively), and 293 K (G, H, I, respectively) were plotted at accumulated simulation time to show the convergence of the MD simulations.

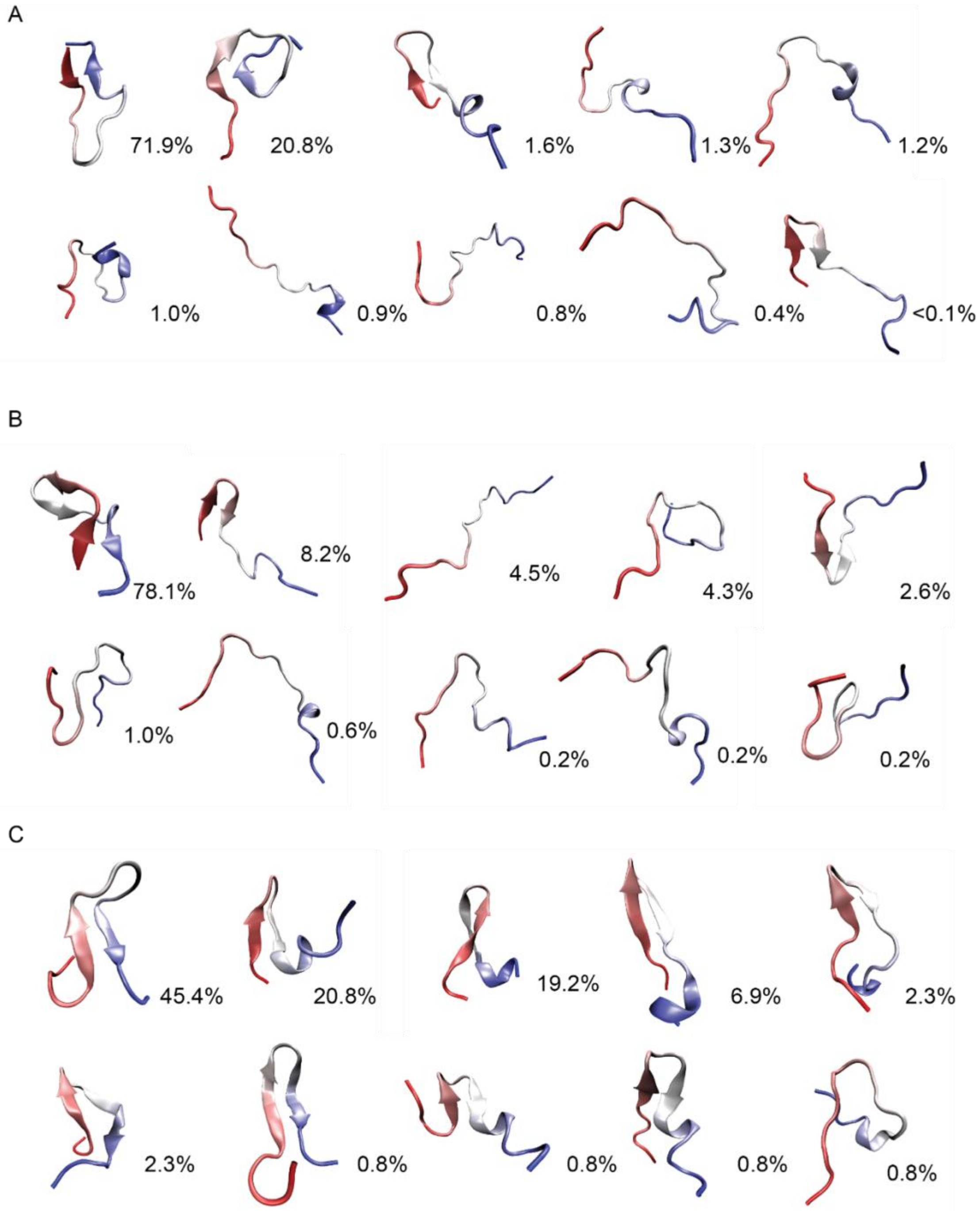

Figure S7 Sample conformations of generated ensembles using CPPTRAJ. CaMKII peptide ensembles are generated by selecting MD trajectory frames from the 293K runs with secondary structure fractions that match the NN-LSQ CD deconvolution results. 10 example structures are produced through Hieragglo clustering of the extracted ensemble using backbone Cα RMS distances. The center structures from 10 generated clusters are shown for (A) RRK, (B) RAK, and (C) AAA to illustrate the effect that each mutation has on the overall conformational behavior. The peptides are colored according to atomic index, from N-terminus (red) to C-terminus (blue), and cluster percentages are shown.

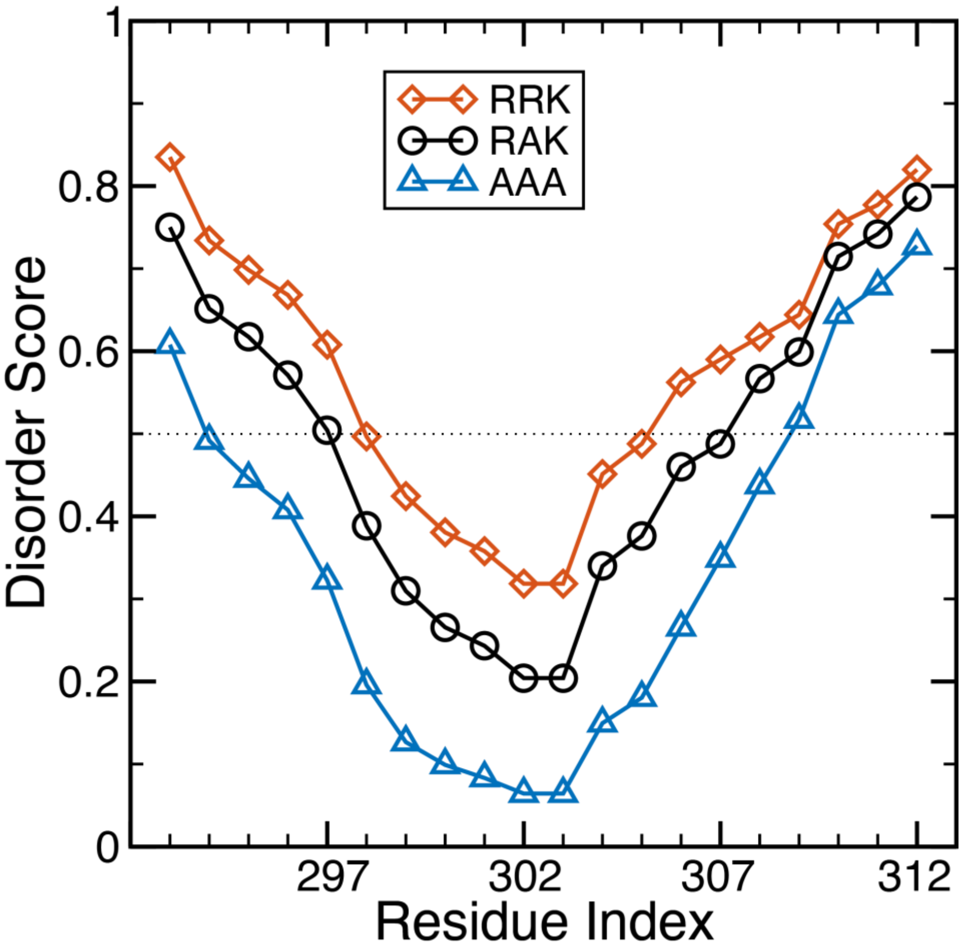

Fig. S8 Computational prediction of unstructured regions in CaMKII peptides using the IUPred web server. The predictor score is plotted against the residue number. The threshold is 0.5 and residues with a higher and lower score are considered to be in disordered and ordered regions, respectively.

Texts:

1. **Equations for CIDER analysis:**

$$\sigma = \frac{(f_+ - f_-)^2}{(f_+ + f_-)}$$

$$\sigma_i = \frac{(f_+ - f_-)_i^2}{(f_+ + f_-)_i}$$

$$\delta = \frac{\sum_{i=1}^{N}(\sigma_i - \sigma)^2}{N}$$

$$\kappa = \frac{\delta}{\delta_{max}}$$

where,
σ is the overall charge asymmetry;
f_+/f_- is the fraction of positively/negatively charged residues;
σ_i is the charge asymmetry for blob segment *i*;
δ is the squared deviation between the segmented blobs and the overall charge asymmetry;
δ_{max} is the maximum value of δ in all possible sequences for a given amino-acid composition;
N is total number of residues.

2. **Convergence analysis for the MD simulations**

Obtaining a well-sampled MD trajectory is crucial for the success of our proposed IDP ensemble generation method, therefore we ensure a well sampled conformation space through convergence analysis of the MD trajectories.

a. We use clustering analysis (Figure S5) to determine whether the majority of conformations have been sampled in our production runs. The number of clusters generated with respect to simulation time can determine the probably that the system will sample new or previously sampled conformations with additional simulation time. We observed that the change in cluster number with respect to time approaches 0 towards the end of each peptide/temperature trajectory, indicating that the majority of conformations have been sampled. A drawback to this method is that the different clustering cutoffs will change the analysis results. Larger cutoffs will not be able to distinguish minor changes in the backbone structure, resulting in small numbers of clusters and faster convergence. Similarly, smaller cutoffs are too strict and may over separate structures that should be grouped together, erroneously indicating that the trajectory diverges.
b. In addition to clustering analysis, the histograms of the distributions of the potential energy of the peptides at temperatures 277 K, 285 K, and 293 K clearly show convergence was

approached with increasing accumulated time (Figure S6). To quantically determine how the potential energy distributions change with respect to simulation time, we applied Kullback–Leibler (KL) divergence [4, 5] (Table S5). KL divergence between the probability distribution P (reference) and Q is defined as follows,

$$KL(P, Q) = -\sum_{x} P(x) \ln \frac{Q(x)}{P(x)}$$

KL divergence analysis indicates a well-sampled trajectory if the changes between potential energy distributions at different times is small. A value of zero indicates that the two distributions are identical. We systematically calculated the KL divergence between the distributions of accumulated trajectories at a simulation time interval of 0.6 μs. The results of our analysis indicate that all trajectories have reached convergence since the KL divergence between potential energy distributions approach to a small value of 0.01 towards the end of our simulation runs.

3. Clustering extracted peptide ensembles using CATS

Due to the large number of ensemble frames extracted from our MD trajectories, it became necessary to cluster the structures so that identifying features could be possible. In a previous study [6], we developed a clustering algorithm that was designed specifically for IDPs. A requirement of the algorithm is that the trajectory dihedral angle distributions be Gaussian-like. A histogram of the φ and ψ dihedral angles of each peptide residue (40 in total) is generated using a bin size of 3.6°. To reduce noise in the distributions, we use a Gaussian weighted moving average filter to smooth the data. We then fit Gaussian curves to the distributions and input the resulting fitting data into CATS. For our analysis of RRK, RAK and AAA, we used an ε value of 3, and 4-coordinate relaxation for all initial clusters with populations under 10% of the total ensemble size. We chose to display the top 10 clusters for each peptide ensemble, which varied with respect to accumulative size with RRK having the lowest total population and AAA having the highest total population in 10 clusters.

4. Validation of NN-LSQ deconvolution with SDP48 data set

The performance of NN-LSQ, CONTIN/LL, SELCON3, and CDSSTR deconvolution methods using SDP48 data set was evaluated using RMSD (δ) and correlation (r) coefficients defined by Woody and Sreerama [7, 8] based on 411 protein CD spectra obtained from the Protein Circular Dichroism Data Base (PCDDB) [9] with known Protein Data Bank (PDB) entries. To determine the effect of secondary structure content on the performance of each deconvolution method, the performance coefficients are calculated

for subsets of proteins with varying amounts of helix, strand, turn and unordered structure content.

Supplementary References

1. Wiedemann, C., P. Bellstedt, and M. Gorlach, *CAPITO--a web server-based analysis and plotting tool for circular dichroism data.* Bioinformatics, 2013. **29**(14): p. 1750-7.
2. Williams, R.M., et al., *The protein non-folding problem: amino acid determinants of intrinsic order and disorder.* Pac Symp Biocomput, 2001: p. 89-100.
3. Daura, X., et al., *Peptide folding: When simulation meets experiment.* Angewandte Chemie-International Edition, 1999. **38**(1-2): p. 236-240.
4. Kullback, S. and R.A. Leibler, *On Information and Sufficiency.* Ann. Math. Statist., 1951. **22**(1): p. 79-86.
5. Eguchi, S. and J. Copas, *Interpreting Kullback–Leibler divergence with the Neyman–Pearson lemma.* Journal of Multivariate Analysis, 2006. **97**(9): p. 2034-2040.
6. Ezerski, J.C. and M.S. Cheung, *CATS: A Tool for Clustering the Ensemble of Intrinsically Disordered Peptides on a Flat Energy Landscape.* J Phys Chem B, 2018. **122**(49): p. 11807-11816.
7. Sreerama, N. and R.W. Woody, *Estimation of protein secondary structure from circular dichroism spectra: comparison of CONTIN, SELCON, and CDSSTR methods with an expanded reference set.* Anal Biochem, 2000. **287**(2): p. 252-60.
8. Sreerama, N., S.Y. Venyaminov, and R.W. Woody, *Estimation of protein secondary structure from circular dichroism spectra: inclusion of denatured proteins with native proteins in the analysis.* Anal Biochem, 2000. **287**(2): p. 243-51.
9. Whitmore, L., et al., *PCDDB: the Protein Circular Dichroism Data Bank, a repository for circular dichroism spectral and metadata.* Nucleic Acids Res, 2011. **39**(Database issue): p. D480-6.
10. Dosztanyi, Z., et al., *IUPred: web server for the prediction of intrinsically unstructured regions of proteins based on estimated energy content.* Bioinformatics, 2005. **21**(16): p. 3433-4.
