# Supplementary material for: Molecular Dynamics Ensemble Refinement of Intrinsically Disordered Peptides According to Deconvoluted Spectra from Circular Dichroism
Source: Biophys J. 2020 Feb 25;118(7):1665–78. doi: 10.1016/j.bpj.2020.02.015 (PMC7136346; doi:10.1016/j.bpj.2020.02.015)
Supplement: Document S2. Article plus Supporting Material [file mmc2.pdf]

Biophysical Journal
Article

Biophysical Society

Molecular Dynamics Ensemble Refinement of Intrinsically Disordered Peptides According to Deconvoluted Spectra from Circular Dichroism

Jacob C. Ezerski,[1] Pengzhi Zhang,[1] Nathaniel C. Jennings,[1] M. Neal Waxham,[2] and Margaret S. Cheung[1,3,*]
[1]Department of Physics, University of Houston, Houston, Texas; [2]Department of Neurobiology and Anatomy, University of Texas, Health Science Center at Houston, Houston, Texas; and [3]Center for Theoretical Biological Physics, Rice University, Houston, Texas

ABSTRACT We have developed a computational method of atomistically refining the structural ensemble of intrinsically disordered peptides (IDPs) facilitated by experimental measurements using circular dichroism spectroscopy (CD). A major challenge surrounding this approach stems from the deconvolution of experimental CD spectra into secondary structure features of the IDP ensemble. Currently available algorithms for CD deconvolution were designed to analyze the spectra of proteins with stable secondary structures. Herein, our work aims to minimize any bias from the peptide deconvolution analysis by implementing a non-negative linear least-squares fitting algorithm in conjunction with a CD reference data set that contains soluble and denatured proteins (SDP48). The non-negative linear least-squares method yields the best results for deconvolution of proteins with higher disordered content than currently available methods, according to a validation analysis of a set of protein spectra with Protein Data Bank entries. We subsequently used this analysis to deconvolute our experimental CD data to refine our computational model of the peptide secondary structure ensemble produced by all-atom molecular dynamics simulations with implicit solvent. We applied this approach to determine the ensemble structures of a set of short IDPs, that mimic the calmodulin binding domain of calcium/calmodulin-dependent protein kinase II and its 1-amino-acid and 3-amino-acid mutants. Our study offers a, to our knowledge, novel way to solve the ensemble secondary structures of IDPs in solution, which is important to advance the understanding of their roles in regulating signaling pathways through the formation of complexes with multiple partners.

SIGNIFICANCE It is challenging to experimentally determine the structural ensemble of an intrinsically disordered peptide (IDP) alone because it lacks a defined structure in solution. Herein, we have developed a computational method of atomistically refining the structural ensemble of IDPs from the experimental measurement by circular dichroism. Our study offers a, to our knowledge, novel way to solve the secondary structures of the IDPs in solution, which is important to advance the understanding of their roles in regulating signaling pathways through the formation of complexes with multiple partners.

INTRODUCTION

Intrinsically disordered proteins/peptides (IDPs) are a category of proteins that possess a poorly defined equilibrium structure; they sample an ensemble of weakly ordered and unordered structures in solution (1–5). IDPs have been shown to play a central role in biological systems through cellular signaling, regulation, and translation (4,6,7). Additionally, misregulated IDPs are associated with cancer (8) and neurodegenerative diseases (9–11) such as Alzheimer's disease. A distinguishing feature of IDPs is that they do not adhere to the classical structure-function paradigm and typically form stable secondary or tertiary structures only upon binding to target proteins (12,13). The lack of stable structures in the ensemble of unbound state (14–17) enables binding to multiple targets on demand while maintaining a degree of selectivity and specificity because of their polymorphic properties (18). Multiple binding pathways exist between a given IDP and its protein targets (19). Furthermore, IDPs are susceptible to post-translational modifications (20–23).

It is challenging to determine the structural feature from an ensemble of IDPs. Popular methods for experimental

Submitted October 30, 2019, and accepted for publication February 18, 2020.

*Correspondence: mscheung@uh.edu

Editor: Alan Grossfield.
https://doi.org/10.1016/j.bpj.2020.02.015

© 2020 Biophysical Society.
This is an open access article under the CC BY license (http://creativecommons.org/licenses/by/4.0/).

structure determination of proteins, such as cryogenic electron microscopy or crystallography, are incapable of determining the structure of IDPs (12). Solution experimental methods such as NMR spectroscopy are only able to produce an ensemble-averaged structure; thus, additional analysis must be performed to generate the structural ensemble (24). Computational approaches such as molecular dynamics (MD) simulations are also used to generate IDP structures; however, these methods largely rely on Hamiltonians whose coefficients are tuned using experimentally determined structures of stable proteins, resulting in overly biased structures (25–27). To address these drawbacks, combined computational and experimental approaches have also been used (16,28–30). A necessary feature of these combined approaches is the conversion between experimental observables and computationally generated structures. NMR structure back calculations (31) use a database that provides a relationship between known structures and chemical shifts. Unfortunately, the NMR chemical shift databases consist of conformationally stable proteins with α-helix and β-sheet structures instead of IDPs. The relationship between the spectroscopic observables and the distinguishing feature of a given protein is deconvoluted from the set of reference structures so that the features of proteins with unknown structures can be determined. This is the typical method for generation of computational models and force field refinement using spectroscopic methods (29,31–35). Despite the popularity of NMR analysis, there are several advantages to using circular dichroism (CD) spectroscopy for the analysis of IDPs in certain circumstances. CD measurements are of low cost, can be quickly performed, and require a small amount of sample material (36,37); however, they cannot provide high-resolution (residue-specific) structure approximations.

We used several standard CD deconvolution algorithms, including SELCON3, CDSSTR, and CONTIN/LL (38), and the reference data set SDP48 (39) to analyze our IDP experimental CD spectra. We discovered incoherent outcomes on measuring IDPs, mostly likely due to biases from these algorithms that favor defined secondary structures from stable globular proteins. Another deconvolution algorithm with no such biases, developed in the 1980s, uses a non-negative least-squares (NN-LSQ) fitting method for solving globular structures (40). However, the reference data set the authors used then does not include any information from denatured peptides. Now that we have noticed the knowledge gap for solving the CD spectra from IDPs, we applied NN-LSQ in conjunction with the SDP48 data set developed in the 2000s to infer secondary structures in our study. The results suggest that the NN-LSQ method in conjunction with the SDP48 reference data set is superior for proteins with high degrees of disorder, prompting us to use the results from this method in subsequent analysis. Using the secondary structure features from our CD deconvolution, we extract an approximate ensemble of structures from all-atomistic MD simulations. We applied this approach on refining the structures of a set of small disordered peptides derived from the calmodulin (CaM)-binding domain of calcium/CaM-dependent protein kinase II (CaMKII, 293–312) and its 1-amino-acid and 3-amino-acid mutants (see Table 1 for the amino acid sequences). These peptides were chosen for detailed examination because the mutated peptide induces a significant (up to 3000-fold) decrease in the CaM binding equilibrium dissociation constant (K_d) in solution at physiological ionic strength (41), with no current understanding of the underlying mechanism.

With our combined approach of CD experiments and MD simulations, we have unexpectedly discovered that the increase of secondary structures in a particularly revealing peptide mutant (AAA) was due to the formation of a β-hairpin conformation, which we speculate is the mechanism behind changes in the encounter rate for this set of IDPs. Obtaining the structural ensembles of CaMKII peptides was a necessary and essential step toward a more accurate estimation of their binding rates for CaM and presently serves as a, to our knowledge, novel example for how secondary structure can be a barrier to productive protein-protein interactions. The deconvolution of CD spectra and subsequent refinement of MD data associated with IDPs and proteins with significant disorder is extremely useful for studying the all-atom conformational dynamics of IDPs, which continue to remain elusive.

MATERIALS AND METHODS

Peptide synthesis and preparation

The three 20-amino-acid-long peptides used in this study were modeled after the CaM-binding domain of CaMKII (residues 293–312; see amino acid sequences in Table 1) and were synthesized by LifeTein LLC (Somerset, NJ). Their purity was greater than 95%, and the composition of each peptide was validated by mass spectroscopy. The kinetics of their binding to calcium/CaM was determined previously using stopped-flow fluorimetry (41), and the values for k_{on} and k_{off} can also be found in Table 1. These experiments revealed that mutation (R296A/R297A/K298A) produced a 3000-fold decrease in the binding affinity.

Measurement with CD spectroscopy

Far-ultraviolet CD spectra were collected on a JASCO-815 spectrophotometer (Easton, MD) controlled by Spectra Manager software. Suprasil cuvettes with a 1.0 mm pathlength were used for all experiments. The spectrometer parameters were typically set to the following unless noted otherwise: bandwidth, 1 nm; response time, 1 s; and data pitch, 0.2 nm/min. A solution consisting of 100 μM peptide was made using 10 mM

TABLE 1 CaMKII Peptide Sequences Are Shown with Mutated Residues in Bold

Peptide	Sequence
RRK (wild-type)	$_{293}$FNARRKLKGAILTTMLATRN$_{312}$
RAK (mutant: 1 site)	$_{293}$FNAR**A**KLKGAILTTMLATRN$_{312}$
AAA (mutant: 3 sites)	$_{293}$FNA**AAA**LKGAILTTMLATRN$_{312}$

Tris buffer (pH 7.5), and measurements were taken by scanning the excitation wavelength between 190 and 260 nm with temperature controlled at 20°C. A total of 10 data accumulations for each run were made with a sweep rate of 100 nm/min. Data collection was repeated for each peptide a total of three times, using a freshly prepared sample in each run.

Deconvolution of CD using a standard package

The CDPro software package suite (38,42) was used to deconvolute the experimental CD spectra of the wild-type and mutant CaMKII peptides. We used the soluble and denatured protein (SDP48) data set in conjunction with the CDPro standard numerical fitting methods: CDSSTR, CONTIN/LL, and SELCON3 (38,42). Because CDPro gives reliable results with CD data in the range of wavelengths 190–240 nm when a large reference set is used (such as SDP48) (38), we inputted our data in the same range in increment of 1 nm. The resulting structure approximation is presented as fractional values for six main secondary structure categories: helix (regular), helix (distorted), strand (regular), strand (distorted), turn, and unordered. We generalized the secondary structure codes into four main categories by consolidating the helix (regular) and helix (distorted) into the helix category and strand (regular) and strand (distorted) into the strand category for comparison of the structure fractions produced by other analysis methods (see Table 2).

Deconvolution of CD data using NN-LSQ fitting

To reliably deconvolute the experimentally determined CD spectra of the wild-type and mutant CaMKII peptides, we applied an NN-LSQ fitting method (40). Unlike other tools using generalized spectra for each secondary structure, we used the reference data set SDP48, which consists of CD spectra of 48 soluble and denatured proteins, as the basis spectra. We assumed that a linear combination of CD spectra from the reference data set is sufficient to approximate the experimental spectra seen in RRK, RAK, or AAA and that the CD spectra of the reference proteins are linearly independent. The squared difference between any non-negative linear combination of the CD basis spectra and the experimental CD spectrum Δ^2 is minimized by finding the optimal weight coefficients $\vec{x}$, as shown in Eq. 1.

$$\Delta^2 = \left\| \boldsymbol{C} \cdot \vec{\boldsymbol{x}} - \vec{\boldsymbol{b}} \right\|^2, \tag{1}$$

TABLE 2 Consolidation of CDPro and DSSP Structure Annotations into Generalized Helix, Strand, Turn, and Unordered Categories

Defined Structure Categories	CDPro Structures	DSSP Structures	CPPTRAJ Implementation of DSSP
helix	helix (regular)	α-helix	α-helix
	helix (distorted)	3–10 helix	3–10 helix
β-strand	strand (regular)	β-strand	parallel β-sheet
	strand (distorted)		antiparallel β-sheet
turn	turn	turn	turn
		bend	
other	unordered	π-helix	π-helix
		β-bridge	none
		irregular/loop	
		turn (1 residue)	
		bend (1 residue)	

We choose a consolidation scheme similar to Kardos et al. (86), in which the π-helix secondary structure is counted as unordered because of its lack of distinction as a stable secondary structure. The DSSP was implemented using the AMBERTOOLS trajectory analysis software CPPTRAJ, which contains an alternate set of structure codes despite using the DSSP algorithm.

where **C** is the 51 × 48 matrix representing the 51 CD spectrum points for all 48 reference proteins of SDP48, $\vec{x}$ is a vector ($x_i \geq 0$, $i = 1, 2, \ldots, 48$) of the weight coefficients for the reference proteins, and $\vec{b}$ is the 51 by 1 vector of the experimentally measured CD values of the CaMKII peptide in the 190–240 nm wavelength range. The weight coefficients vector $\vec{x}$ is determined by NN-LSQ fitting. To note, here each coefficient is not bounded between 0 and 1 to account for the possible differences in the signal amplitude in our experimental results and the reference data set CD spectrum.

We subsequently use the fitted weight coefficients vector $\vec{x}$ to compute the secondary structure fractions given by Eq. 2,

$$\vec{\boldsymbol{d}} = \boldsymbol{A} \frac{\vec{\boldsymbol{x}}}{\left\| \vec{\boldsymbol{x}} \right\|}, \tag{2}$$

where **A** is the 6 × 48 matrix representing the six possible secondary structure fractions for each of the 48 reference proteins and $\vec{d}$ is the 6 × 1 secondary structure solution for the CaMKII peptide.

The resulting structure approximation is presented as fractional values for six main secondary structure categories: helix (regular), helix (distorted), strand (regular), strand (distorted), turn, and unordered. We generalized the secondary structure codes into four main categories by consolidating the helix (regular) and helix (distorted) into the helix category, and strand (regular) and strand (distorted) into the strand category for comparison of secondary structure fractions produced by other analysis methods (see Table 2).

Validation of NN-LSQ deconvolution results

The performance of NN-LSQ, CONTIN/LL, SELCON3, and CDSSTR deconvolution methods were compared using the root mean square deviation (δ) and correlation (r) coefficients shown in Eqs. 3 and 4, originally defined by Woody and Sreerama (38). The analysis uses subsets of 411 proteins obtained from the Protein Circular Dichroism Data Bank (PCDDB) (43) with known secondary structures and CD spectra. The PCDDB entries for the selected spectra are provided in Table S11.

$$\delta = \sqrt{\frac{\sum_i \left(f_i^{CD} - f_i^x\right)^2}{N}} \tag{3}$$

and

$$r = \frac{N\sum_i \left(f_i^{CD} \times f_i^x\right) - \sum_{ij}\left(f_i^{CD} \times f_j^x\right)}{\sqrt{\left[N\sum_i \left(f_i^{CD}\right)^2 - \left(\sum_i f_i^{CD}\right)^2\right] \times \left[N\sum_i \left(f_i^x\right)^2 - \left(\sum_i f_i^x\right)^2\right]}}, \tag{4}$$

where N is the number of proteins, f_i^{CD} is the structure content obtained from CD deconvolution for structure i, and f_i^x is the known structure fractional content for structure i.

All-atom MD simulations with implicit solvent of the peptides

MD setup and initialization

Because there is no high-resolution solved structure because of the disordered nature of the CaMKII peptides, we built the initial structures for MD simulations using the LEaP module of AMBERTOOLS 14 (44) based only on the amino acid sequences (Table 1). To be consistent with the experimental study (41), the N- and C-termini of these peptides were not capped or modified. All MD simulations were carried out using the package AMBER 14 with the

ff99sb force field (26,44). We used an implicit solvent model with the generalized Born (45–47) approximation and the modified Born radius parameter set mbondi2 (46). We performed energy minimization on the initial structures using 1000 steps of conjugate gradient, followed by 1000 steps of steepest descent algorithms. The minimized structures were brought to the desired temperatures in two steps: heating each minimized structure to 277, 285, or 293 K, followed by a simulated annealing cycle. Simulated annealing was carried out by heating structure coordinates obtained in the previous step to 400 K over a period of 600 ps, followed by cooling to the designated temperature over a period of 600 ps with velocity randomization every 100 ps. All setup runs used a time step of 2 fs. We restrained hydrogen dynamics by employing the SHAKE algorithm (48). We used Langevin dynamics with a collision frequency of 2 ps^{-1} to regulate the temperature (Langevin thermostat); periodically randomizing the velocity distributions was therefore necessary to avoid the synchronization effects associated with Langevin thermostats (49).

MD production runs

We performed all-atomistic implicit solvent simulations for each CaMKII peptide at 277, 285, and 293 K, replicating the operating temperature of the stop-flow kinetics experiment (41), a midpoint temperature, and the operating temperature of the CD measurements, respectively. The production run was performed at the designated temperature for a period of 80 ns with a 2-fs time step. We sampled energy and trajectory data every 4 ps, which was determined through correlation time analysis. All simulation steps from the setup and production runs were repeated an additional 14 times for every temperature and peptide combination, resulting in a total production run simulation time of 2.4 μs (per peptide per temperature). Trajectories were tested for convergence using two approaches: Kullback-Liebler divergence (50,51) between distributions of the potential energy in accumulated simulation time (Table S5) and cluster analysis with respect to simulation time (Fig. S5). Details of convergence analysis can be found in the Supporting Materials and Methods.

Data-guided extraction of all-atom peptide conformation ensembles

Determination of the secondary structure content in MD trajectories

The secondary structure content of the peptides was computed using the CPPTRAJ module of AMBERTOOLS (52), which calculates structure content based on the Dictionary of Secondary Structures of Proteins (DSSP) (53). The results of our structure analysis generated seven possible secondary structure categories per residue: α-helix, parallel β-sheet, antiparallel β-sheet, 3–10 helix, π-helix, turn, and unordered. We consolidated the seven secondary structure categories into four generalized secondary structure categories (see Table 2) and generated a histogram of the structure codes associated with each residue to produce the overall fractional secondary structure values in each trajectory frame.

Refinement of IDP ensemble structures from MD using CD deconvolution data

Using the secondary structure data for each frame of our MD trajectories, we selected pairs of trajectory frames that produce average secondary structure fractions similar to those observed in the CD deconvolution data from our NN-LSQ fitting. For a given peptide trajectory, frames are extracted in pairs if the following equality is satisfied for each structure fraction:

$$\left| \frac{\left(S_i^k + S_j^k\right)}{2} - S_0^k \right| < \phi, \qquad (5)$$

where S_i^k and S_j^k are the fractional values for the k-th structure category (helix, β-sheet, turn, or unordered secondary structure categories) for frames i and j and S_0^k is the structure fraction for category k derived from our NN-LSQ deconvolution results.

Contact map analysis

CD-guided MD structures of the peptides from the CD-refined ensemble were used for contact map analysis. The definitions are described as follows:

1) A contact between residue i and j (at least four residues away) is formed if any atom from residue i is within a cutoff distance of 4 Å of any atom from the residue j.
2) A backbone (side-chain) contact between residue i and j (at least four residues away) is formed if any backbone (side-chain) atom from the residue i is within a cutoff distance of 4 Å of any backbone (side-chain) atom from the residue j. A single hydrogen atom from glycine is considered as its side chain.
3) A hydrogen bonding contact between residue i and j is formed if a donor atom (D) from residue i is within a cutoff distance of 4 Å of an acceptor atom (A) from residue j and the D-H-A angle through a bonding hydrogen (H) is within a cutoff angle of 30°.

RESULTS

CD spectra indicate a distinct secondary structure shift between RRK and AAA

The CD spectra presented in Fig. 1 show the average secondary structure ensembles of RRK, RAK, and AAA peptides: three peptides of identical length exhibiting significantly different binding kinetics with CaM (41). In general, a negative CD band at 220 nm indicates the presence of helical or strand structures, and a negative band at 195 nm corresponds to denatured or disordered structures (54). Here, the experimental data show the existence of a secondary structure in AAA that does not exist in RRK or RAK. These data suggest that each charged residue mutation reduces the disordered content of the peptide's structure ensemble. Overall, the charged residue mutations of R296A/

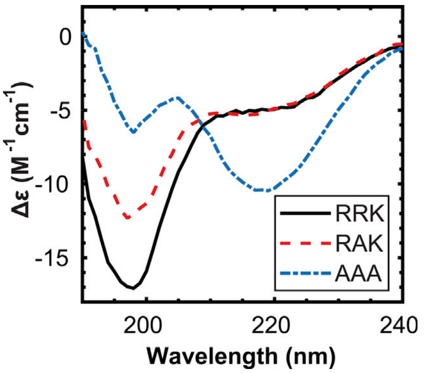

FIGURE 1 Far-ultraviolet CD spectra of the CaMKII peptides. CD spectra were obtained as described in Methodology using a Jasco Model 815 spectrophotometer. A solution consisting of 100 μM of each peptide was made in 10 mM Tris buffer (pH 7.5), and measurements were taken in a 1.0 mm quartz cuvette by scanning the excitation wavelength between 190 and 240 nm with temperature controlled at 20°C. To see this figure in color, go online.

R297A/K298/A result in a significant conformational change from the disorder in RRK to the more ordered structures in AAA.

We first speculated the increased structures in AAA were due to a helical secondary structure because alanine residues have the highest propensity to form α-helices (55). However, the experimental CD spectrum for AAA displays only one negative peak at 222 nm but is missing a second smaller signal peak at 208 nm, which is a hallmark of α-helical regions in CD spectra (54). This indicates that there is a mixture of secondary structure components in the peptides. Therefore, we employed CDPro to deconvolute the CD spectra on the three peptides in the next section.

Standard CD deconvolution solvers produce inconsistent results on the content of secondary structures

We employed three standard CD deconvolution solvers, CDPro, CAPITO, and BeStSeL, in attempts to analyze the structural information of the peptide spectra shown in Fig. 1. We found that all three algorithms show nonconvergence and unacceptably large RMSDs compared with the experimental spectra as follows:

1) CDPro: We generalized the secondary structure codes used by CDPro into four main categories (see Table 2). The three standard deconvolution solvers (CDSSTR, SELCON3, and CONTIN/LL) from CDPro generate inconsistent fractions of secondary structures as shown in Table 3. The CONTIN/LL method shows that RRK contains mostly turn and unordered secondary structures; however, the CDSSTR method shows that RRK contains similar quantities of structured and unstructured regions. In the AAA deconvolution results, the CDSSTR and CONTIN/LL methods suggest opposing secondary structure content, with CDSSTR resulting in the increase of helical fractions and CONTIN/LL resulting in the increase of turn content. The SELCON3 methods appear to perform the worst among the three, giving large RMSDs between the reconstructed CD spectra and the experimental data (Fig. 2) and producing unrealistic fractions of secondary structures.
2) CAPITO: Use of more recently developed tools for the analysis of CD spectra either shows large RMSDs or underestimates the fraction of unordered secondary structure for proteins with rich disordered segments. Specifically, CAPITO (56), which uses basis spectra for each of the α-helix, β-strand, and irregular secondary structures extracted from SP-175, produced a poor fit for the CaMKII peptides (see Fig. S1; Table S1).
3) BeStSel (57) carries out a detailed secondary structure analysis, providing information on eight secondary structure components, and provides improved estimation of the β-strand content. Our analysis of the CaMKII

TABLE 3 Fractional Secondary Structure Approximations Are Given for the CONTIN/LL, SELCON3, CDSSTR, and NN-LSQ Fitted CD Deconvolution Methods

		Helix	Strand	Turn	Unordered	RMSD in $\Delta\varepsilon$
RRK	*SELCON3	0.00	−0.06	−0.07	1.28	15.86
	CDSSTR	0.15	0.32	0.28	0.24	1.38
	CONTIN/LL	0.01	0.01	0.10	0.88	0.35
	NN-LSQ	0.04	0.15	0.09	0.72	0.26
RAK	SELCON3	0.04	0.03	0.01	0.94	4.72
	CDSSTR	0.17	0.29	0.23	0.31	0.84
	CONTIN/LL	0.03	0.02	0.08	0.87	0.40
	NN-LSQ	0.04	0.22	0.13	0.62	0.23
AAA	SELCON3	0.29	0.20	0.19	0.36	3.26
	CDSSTR	0.37	0.30	0.16	0.17	0.63
	CONTIN/LL	0.03	0.05	0.30	0.62	0.44
	NN-LSQ	0.04	0.34	0.17	0.46	0.36

The approximate CD spectrum representing the CaMKII peptides is recreated from a linear combination of SDP48 known conformation and spectra definitions that we developed. The RMSD between the approximated and experimental spectrum ($\Delta\varepsilon$) is given in unit of M^{-1} cm^{-1}.
*SELCON3 was unable to reach a convergent solution during the analysis of RRK.

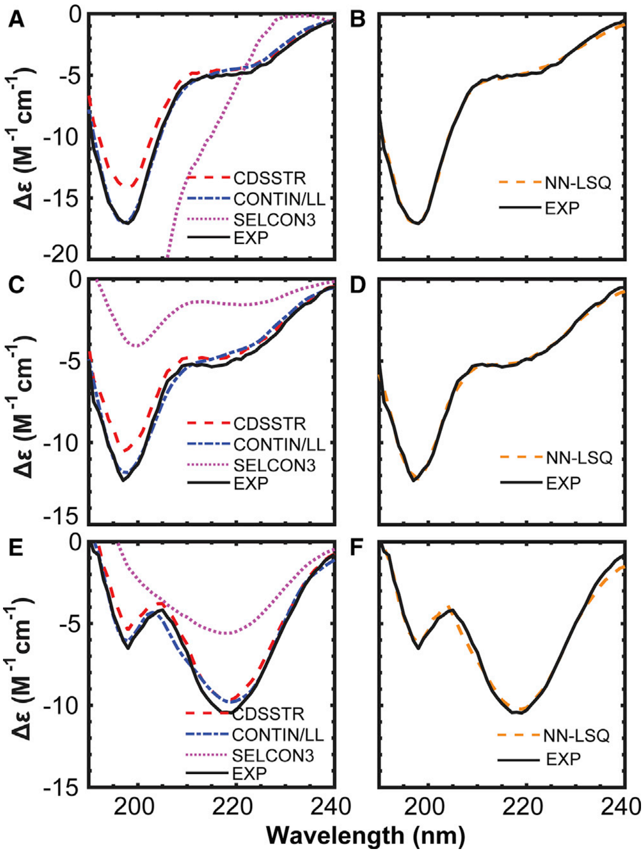

FIGURE 2 Comparison between the fitting of the CD spectra using the CDPro and NN-LSQ fitting. (*A*, *C*, and *E*) The experimental CD spectrum is compared with the calculated CD spectrum derived from the CONTIN/LL, CDSSTR, and SELCON3 methods for RRK, RAK, and AAA peptides, respectively. (*B*, *D*, and *F*) The calculated CD spectrum using the NN-LSQ fitting method and SDP48 data set is compared with the experimental data for RRK, RAK, and AAA peptides, respectively. To see this figure in color, go online.

peptides with BeStSeL produced relatively large RMSDs (Fig. S2) and reinforces that present CD analysis tools are not useful for this class of peptides.

CD deconvolution with NN-LSQ fitting indicates presence of β-hairpin secondary structure

The inconsistencies associated with the standard deconvolution models prompted us to review the fitting methods from the three standard deconvolution solvers. We noted that these methods overly favor helical content by fitting the CD spectrum to a data set of predominantly globular or membrane-bound proteins, as well as by employing algorithms emphasizing the weights on helical structures. To avert these two issues, we chose to fit the CD spectrum with the data set of denatured proteins (SDP48) and search for alternative fitting routines. It is necessary to use the data set of only denatured proteins (rendering the lowest RMSD between the approximated and experimental spectrum ($\Delta\varepsilon$)) because using other data sets made up of globular proteins do not yield good fits (rendering large RMSDs between the approximated and experimental spectrum ($\Delta\varepsilon$)), as shown in Table S2. We used NN-LSQ fitting, which simultaneously took into account the data from all protein structures in the SDP48 reference set and made no a priori assumptions about the secondary structure. Our NN-LSQ fit deconvolution results, presented in Table 3, indicate that the primary effects of the mutation in the CaMKII peptides emerge through an increase in the β-sheet category (strand) secondary structure, whereas the helical content remains the same. The increase in the strand secondary structure is naturally associated with a decrease in disordered secondary structure, where RRK has the highest disordered content with 72%, and AAA has the lowest disordered content with 46%.

All-atom MD simulations produce strongly biased structure ensembles

To generate an equilibrium ensemble of structures for the three peptides, we employed all-atom MD simulations with implicit solvent at three temperatures: 277, 285, and 293 K. A total of 2.4 μs of data sampled at 4-ps intervals was collected for each peptide and temperature combination and analyzed for their secondary structure content using the DSSP. Data produced from this analysis were translated into a four-category generalized secondary structure scheme shown in Table 2. The secondary structure fractions for each trajectory were first averaged to illustrate the overall conformational trend produced in each simulation (Fig. 3). The analysis of the secondary structures shows that the MD simulations was incapable of generating an ensemble of structures that match with the CD analyses. More specifically, compared with the deconvoluted secondary structure fractions from the CD data, the MD ensembles illustrate a significant bias toward helical content. The data for all peptides at all temperatures show the β-sheet content at less than 5% and the helical, turn, and unordered content in the range of 30–40%. Additionally, the DSSP analysis indicates no significant overall secondary structure shift between the wild-type and mutant peptides for all three temperatures. This finding is in contrast to the deconvolution data in NN-LSQ fitting, in which the fraction of β-sheet content for RRK is 15% and increases to 22% and 34% for RAK and AAA, respectively. In summary, the outcomes from the MD simulations do not appear to accurately represent the secondary structure shift that occurs between mutant peptides as indicated by our CD data.

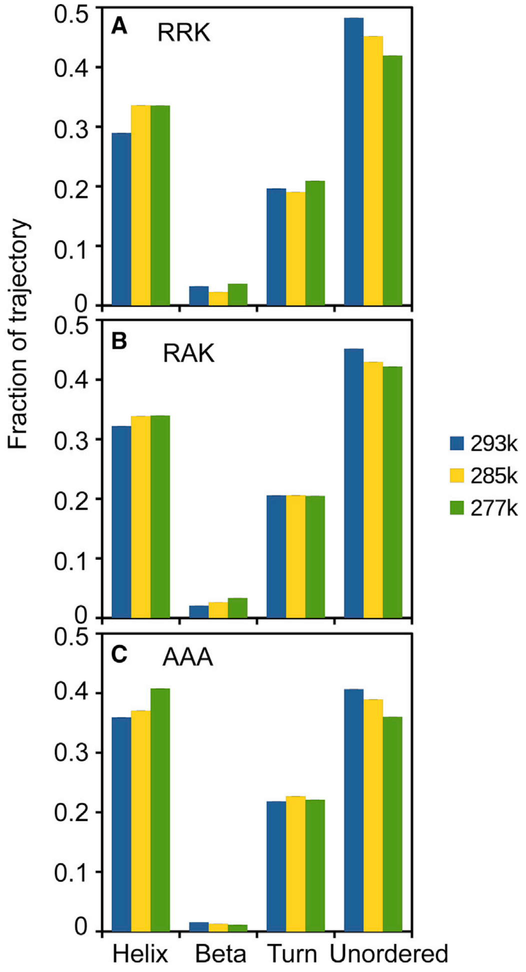

FIGURE 3 Average secondary structure fractions produced by the all-atom CaMKII peptide simulation. Histograms of the secondary structures produced by DSSP analysis for each frame of the CaMKII peptide trajectories are shown for (*A*) RRK, (*B*) RAK, and (*C*) AAA at 277, 285, and 293 K. To see this figure in color, go online.

Approximate structure ensemble of IDPs from all-atom trajectories and CD deconvolution

To gain useful information from the MD simulations that agrees with our CD deconvolution data, we select pairs of

trajectory frames from the production run with similar averaged secondary structure fractions as those observed in our NN-LSQ fitted CD deconvolution data shown in Table 3. We analyzed peptide trajectories for the 293 K production run using a ϕ-value of 0.035 for each structure category (Eq. 5). Using the criteria, we obtained 11,002 structures for RRK, 2410 structures for RAK, and 130 structures for AAA. Deviations between the number of structures generated for each peptide appears to be correlated with the relative β-sheet content. All MD trajectories displayed poor sampling of β-sheet structures (Fig. 3), which may explain the decreasing number of extracted frames as the β-sheet content for each peptide increases. Specifically, $\sim$5% of the sampled trajectory frames contained β-sheet secondary structures for all three peptides. The results from NN-LSQ deconvolution showed that RRK, RAK, and AAA contained 15, 22, and 34% β-sheet secondary structures, respectively (Table 3). Because RRK contained the lowest fraction of β-sheet content, significantly more frames were able to be extracted from our trajectories than for AAA using Eq. 5.

We assume that the structural ensemble of the MD simulations is biased but still samples the correct peptide conformations in significantly smaller quantities. Because spectroscopic methods produce observables corresponding to the ensemble-averaged state, we only require that the extracted MD frames produce an ensemble whose average corresponds to the experimental CD data. Using the solutions obtained from CD deconvolution enables us to separate MD trajectory data that agree with the experimental data from biased trajectory data.

A set of 10 structures representing each peptide ensemble was generated by clustering. Initially, the Hieragglo clustering method from CPPTRAJ with 10 total clusters was used. The results of this clustering method appear to be misleading because of the disproportionally large populations of the first clusters in RRK and RAK (Fig. S7; Table S6). Because the CaMKII peptides possess significant fractions of disordered content, it is likely that these large clusters have conformational variation within them and are poor representations of the ensemble. To gain better resolution of the representative ensemble structures, a previously developed clustering algorithm was chosen to resolve the extracted structures. The combinatorial averaged transient structure (CATS) method has produced better structure resolution for IDPs than traditional clustering methods (58) and is therefore employed in this study.

The selected structures (Fig. 4) from CATS represent a set of highly probable conformations exhibited by the peptides in solution. Based on these representative structures, RRK and RAK display significant conformational variation compared with AAA, which forms compact β-sheet structures. In our NN-LSQ CD deconvolution results, RRK and RAK present a high percentage of unordered structure at 72 and 62%, respectively. On the other hand, AAA possesses a lower degree of unordered structure at 46% (Table 3). This

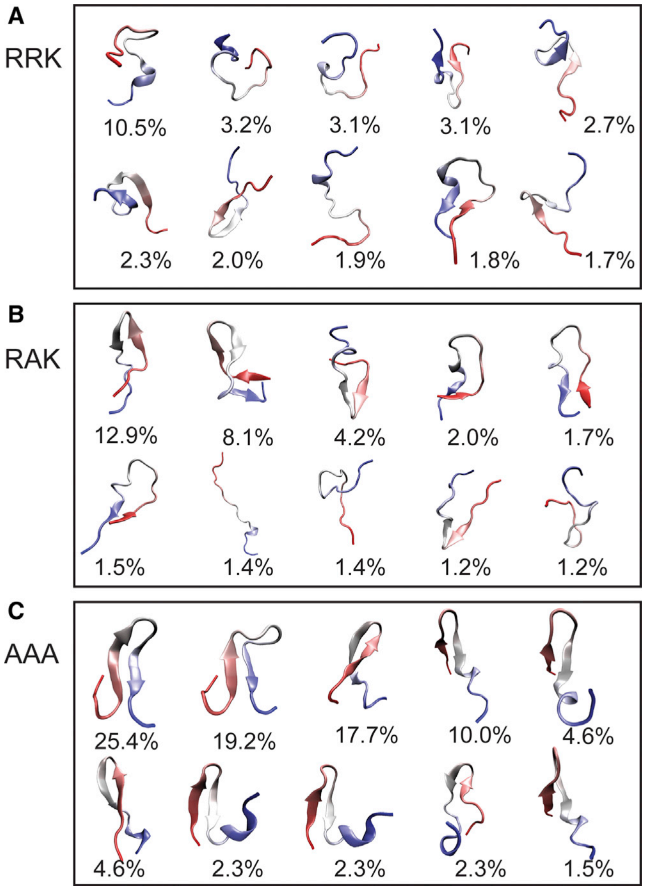

FIGURE 4 Sample conformations of generated ensembles. CaMKII peptide ensembles were generated by selecting MD trajectory frames from the 293 K runs with secondary structure fractions that match the NN-LSQ CD deconvolution results. To illustrate structure features, the generated ensembles are clustered using an algorithm designed to cluster IDPs developed from our group: CATS (58). Details about this clustering method can be found in the Supporting Materials and Methods. Center structures from the 10 most populated clusters are shown for (*A*) RRK, (*B*) RAK, and (*C*) AAA. The peptides are colored according to atomic index, with the N-terminus shown in red and the C-terminus shown in blue. To see this figure in color, go online.

result is consistent with the generated structure ensemble, which possess a maximal RMSD of 12.5 Å for RRK, 12.4 Å for RAK, and 10.5 Å for AAA (Table S3). The RMSD analysis of the generated ensemble also illustrates that AAA has the lowest standard deviation of RMSD values (1.0 Å) compared with RRK and RAK (1.4 and 1.7 Å, respectively).

An increasing secondary structure content can be observed in Fig. 4 as a result of each sequential mutation of the RRK peptide. This observation is in agreement with the shift in ordered and disordered content predicted by CD deconvolution, despite the apparent force field bias observed in the analysis of the complete trajectories (Fig. 3). We acknowledge that the precise quantitative shift in secondary structure fractions in each mutant may not be completely represented by the generated ensembles shown in Fig. 4; however, they illustrate the approximate location of residual secondary structure.

The set of RRK structures (Fig. 4 *A*) contains relatively small regions of helical and β-hairpin regions. The N-terminus appears to be largely unstructured, with the ability to participate in β-strand formation with C-terminus residues. On the other hand, the C-terminus appears to form turn, helical, and hairpin structures more readily with other C-terminal or central residues. In the set of RAK structures (Fig. 4 *B*), the presence of β-strand conformations is more prevalent in comparison with RRK. It can be observed that the N-terminus of RAK participates in the majority of β-hairpin structure formation. Additionally, the number of structures with turn/helical regions in the C-terminus has decreased with respect to RRK; however, this appears to be correlated with the increase in β-hairpin structure formation. Lastly, the set of AAA structures (Fig. 4 *C*) all contain the β-hairpin secondary structure; however, there appear to be two distinct variations of the hairpin: a symmetrical β-hairpin structure and an asymmetrical hairpin-helix structure. The asymmetrical structures begin their hairpin motif closer to the N-terminus and form a helical structure on the unbound C-terminal tail. Alternatively, the symmetrical structures start forming the hairpin motif in the central region of the peptide, with N- and C- terminal binding instead.

Contact map analysis shows AAA mutant adopts strong secondary structure formation

To gain more insight in the characteristics of the differential hairpin structures in the three peptides, we analyzed the amino acid contacts formed by each peptide (Fig. 5). The CaMKII peptide can be broken down into three regions: N-terminus, C-terminus, and the center. The N-terminal region (293–298) contains positively charged residues in RRK/RAK and neutral residues in AAA. The central region, or the CaM-binding motif (L299–L308), is mainly composed of hydrophobic residues. The C-terminal region of each peptide (309–312) contains a charged arginine residue, which can potentially form hydrogen bonds or repel other positively charged residues in the N-terminus.

1) In the wild-type peptide RRK, as seen in Fig. 5 *A*, the probability of contact formation is generally low (<0.5), which suggests high variation in the conformations adopted by the peptide. Secondary structures such as β-sheets can be formed at a low probability. More specifically, the N-terminus and the C-terminus can possibly form an antiparallel β-sheet, suggested by the interactions in the cross-diagonal region of the contact map (*blue ellipses*), especially between side chains of M307 and the middle basic residue (R297); the central region of the peptide can form parallel β-sheets, suggested by the low-probability (∼0.2) interactions in the region of the contact map that are parallel to the diagonal (*orange ellipse*); more likely, the central region can form an α-helix, indicated by the sparsely distributed higher-probability contacts (∼0.4) parallel to the diagonal (residues separated by four residues, *dotted lines* parallel to the *diagonal* in Fig. 5 *A*), such as the backbone-to-backbone contact between L304 and L308 and the side-chain-to-side-chain contacts between L299 and I303 and between I303 and M307.
2) Upon mutation of R297A, in Fig. 5 *B*, the interactions are sparser but mostly of higher probabilities. Compared with the wild-type, there is a higher probability of forming an antiparallel β-sheet between the N-terminus and the central region of the peptide (*blue ellipses* in Fig. 5 *B*). The N-terminus is likely to form stable contacts with hydrophobic residues in the central region close to the C-terminus, especially between the residues around the mutation A297 and M307-L308. Compared with the wild-type, interactions in the center of the RAK peptide do not seem to form any parallel β-sheet structures (Figs. 5 and S3).
3) In the peptide AAA, further compaction in the peptide structure (Fig. 4) and increase in the secondary structures are observed (Fig. 5 *C*). In contrast to RRK and RAK, there is a relatively high probability of forming antiparallel β-sheet structures between the N-terminus and the central region (*blue ellipses*, Fig. 5 *C*) and a low probability of forming antiparallel β-sheet structures between the central region and the C-terminus (*orange ellipses*, Fig. 5 *C*). Interestingly, the mutated residues play an essential role. There are stable backbone-to-backbone interactions between the hydrophobic region formed by the mutated residues and neighboring residues (A297–G301) and hydrophobic residues in the central region (M308–L309) and side-chain-to-side-chain interactions between the mutated residues and residues in the central region, as well as the C-terminus. To note, the mutated residue A298 has a high probability for forming a side-chain-to-side-chain contact with charged residue R311, which is prohibited in RRK or RAK because of electrostatic repulsion. In summary, the AAA peptide shows a high probability of adopting an antiparallel β-sheet conformation (as shown in Fig. 4 *C*), and the stabilizing hydrophobic interactions of the AAA mutant may interfere with helix formation, which is a necessary conformational adjustment that aligns the CaM-binding motif to residues in CaM, including residues L299, I303, and L308 (lack of interactions within the CaM-binding motif along the *lines* parallel to the *diagonal* in Fig. 5 *C*).

Furthermore, we analyzed the hydrogen bonds within each peptide ensemble to investigate the role of charged residue distribution in each peptide's equilibrium conformation (Fig. 6). Our analysis reveals two diagonal hydrogen bonding patterns in AAA between N- and C-terminal residues that do not exist in RRK or RAK. Upon closer examination of AAA, we observe that the charged residue mutation sites form hydrogen bonds with the C-terminal region near R311. This binding pattern appears to contribute to the β-sheet secondary structure

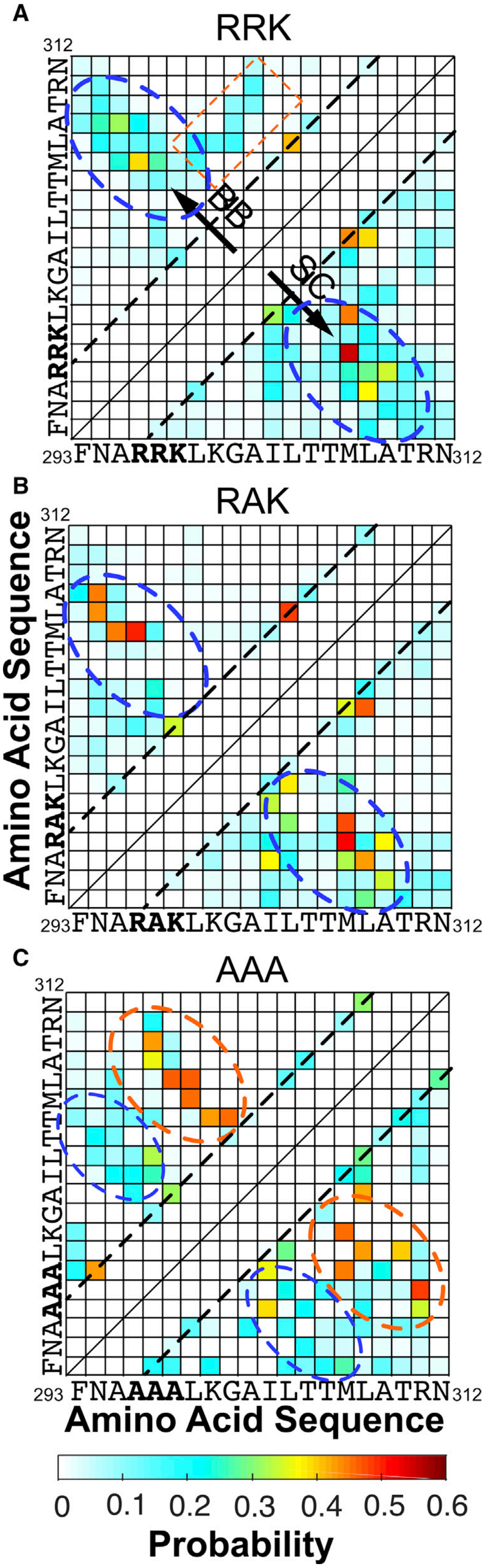

FIGURE 5 Contact probability map of the CD-refined MD structures. Probabilities of contact formation are plotted for peptides (*A*) RRK, (*B*) RAK, and (*C*) AAA. The upper triangle and lower triangle depict the probability of backbone-to-backbone (BB) and side-chain-to-side-chain (SC) contact formation, respectively. The amino acid sequences are provided as the axis labels. The blue and orange ellipses encircle antiparallel β-sheet structures, the orange rectangle encloses a parallel β-sheet structure, and the dotted straight lines mark the contacts in the α-helical structures. The criteria of the contact formation are defined in the Materials and Methods. To see this figure in color, go online.

formation of AAA. On the other hand, the two highest-probability contacts exist between T310-M307 and R311-L308, which may contribute to the formation of the C-terminal helical motif that is observed in several extracted AAA ensemble conformations (Fig. 4 *C*). RRK and RAK show alternate hydrogen bond patterns on the diagonal that loosely resemble helical or turn conformations. RRK and RAK appear to form only one high-probability hydrogen bond between M307 and L304, which is not formed by AAA. Although RRK and RAK both appear to form low-probability hydrogen bonds with both N- and C-terminal residues, RAK possesses a high-probability hydrogen bond between R296 and M307. Examination of the bonds formed by the charged residues of the N-terminus in RRK and RAK illustrates a pattern of interactions with over half of the other residues, with RRK possessing a greater spread of low-probability bonds than RAK. Direct binding pattern changes between RRK, RAK, and AAA at the mutation sites are expected; however, many of the new hydrogen bonds do not appear to directly involve the charge residues at the mutation sites, implying the effect of charged residue mutations is not localized. This phenomenon is observed in the L299-L308 hydrogen bond: AAA has a high probability of forming this contact compared to RRK and RAK, even though neither residue was mutated.

DISCUSSION

Conformational ensemble of the CaMKII peptides are dependent on charged residue distribution

Our experimental CD measurements and CD deconvolution results indicate that the residual secondary structure of the three-residue mutant AAA is hairpin like. Additionally, our analysis revealed that the RRK and RAK peptides were composed of disordered and hairpin conformations, along with 4% residual helix structure (Table 3). The equilibrium conformational ensemble shift between the wild-type and mutant CaMKII peptides is directly correlated to solvation and electrostatic effects. Previously, several studies have shown that the specific distribution of charged residues within a peptide will affect the equilibrium conformation (59–61). To determine whether the conformational shift observed between RRK, RAK, and AAA can be attributed to changes in charge distribution, we analyzed the sequences of the CaMKII peptides using the IDP analysis tool CIDER (62). Our analysis found that AAA is predicted to be in a compact or globular ensemble, whereas RRK is predicted to be in the most expanded conformation (see Fig. S4; Table S4). This result was expected because RRK has the most heterogeneously distributed charges with respect to RAK or

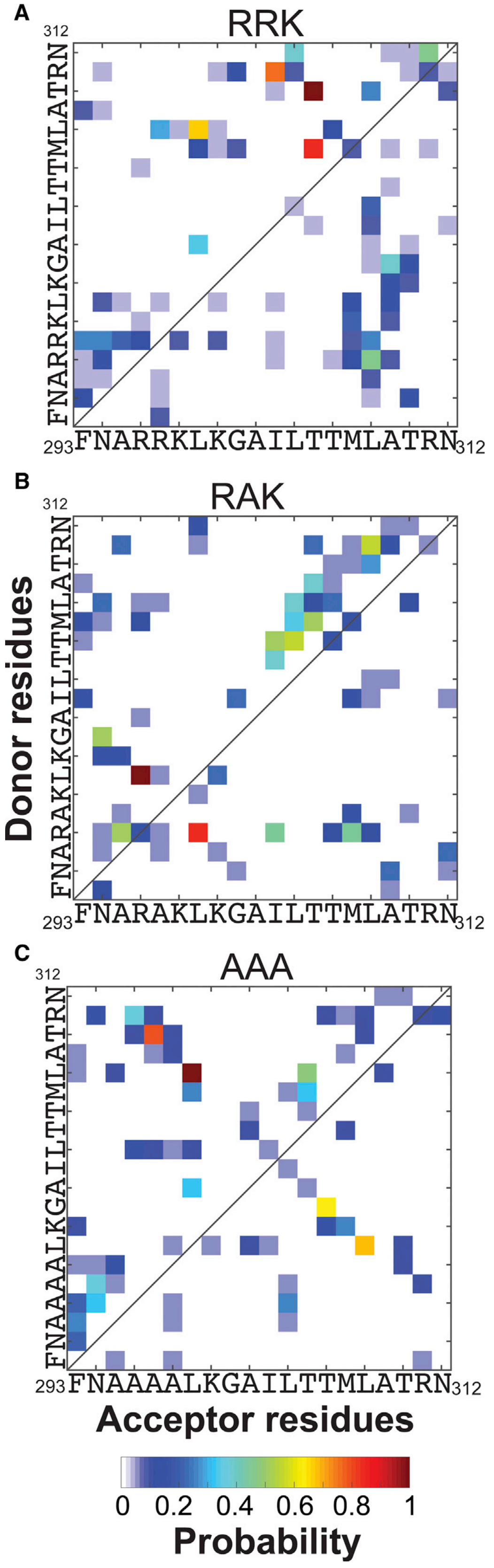

FIGURE 6 Hydrogen bond probability map. The relative probability of intramolecular hydrogen bond formation is shown for the ensemble of structures extracted from all-atom MD simulations using the results from NN-LSQ CD deconvolution for (*A*) RRK, (*B*) RAK, and (*C*) AAA. Contacts are defined using a 30° angular cutoff and 4 Å distance cutoff between hydrogen bond donor and acceptor residues. Contact probabilities are scaled such that the highest contact probability is 1. To see this figure in color, go online.

AAA. CIDER also predicted that RAK will be in a globular form based on the fraction of charged residues; however, the similarity between the RRK and RAK CD spectra leads us to question the validity of this prediction.

NN-LSQ deconvolution yields best results for proteins with large disordered regions

We chose to employ an alternative deconvolution method for the CaMKII peptide because using the NN-LSQ algorithm in conjunction with a data set containing soluble and denatured proteins (SDP48) yielded the best results. Table 3 illustrates that the reconstructed CD spectra using NN-LSQ has the lowest RMSD with respect to the experimental spectra in the 190–240 nm region for all three CaMKII peptides. Additionally, we searched PCDDB (43) to obtain a set of proteins with known structures and associated CD spectra. This criterion matched a set of 411 proteins, which were used to validate the NN-LSQ algorithm results. We determine the quality of deconvolution and validate our results using the methods described by Sreerama and Woody for SELCON3, CDSSTR, and CONTIN/LL (38,39), which are given by Eqs. 3 and 4. We analyzed subsets of proteins with varying disordered content, which showed that the NN-LSQ method has the highest correlation and lowest deviation between predicted and known secondary structures for proteins with high degrees of disorder (Fig. 7). Conversely, our validation revealed that the CDPro algorithms perform better than the NN-LSQ method for proteins that contain lower fractions of disordered content. The complete validation and comparison results can be found in Tables S7–S10.

The CONTIN/LL, CDSSTR and SELCON3 fitting algorithms have been developed to reliably analyze the spectra of stable proteins through a robust set of iterative and variable selection rules that can discard certain solutions. Because the algorithm features were optimized for globular proteins, the accepted solutions are inherently biased and are not applicable for this set of CaMKII peptides, which have a high probability of disorder (Fig. S8; Table S4). Because of this revealed incompatibility, we deconvoluted the experimental CD spectra with NN-LSQ and the SDP48 reference protein set.

Force fields for MD simulations favor helical formation

The Hamiltonian used in MD force fields refines coefficients through experiments with larger globular proteins, which are structured by nature (63,64). This effect has been

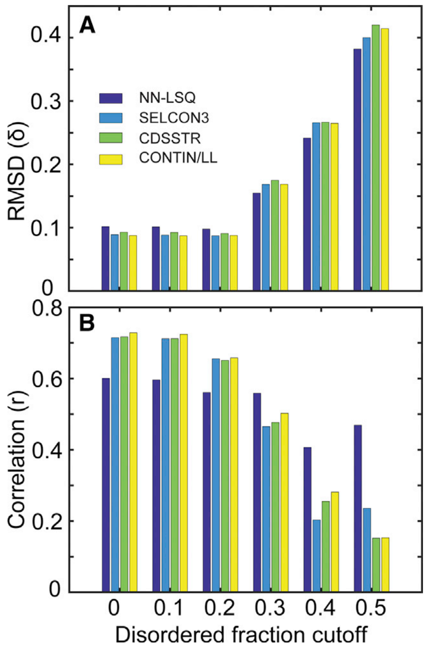

FIGURE 7 Validation indices of deconvolution fitting. (*A*) The RMSD (δ) and (*B*) the correlation (r) between deconvoluted structure and known structure fractions are calculated using Eqs. 3 and 4 for the SELCON3, CONTIN/LL, CDSSTR, and NN-LSQ methods using protein sets with varying unordered structure content. A cutoff of 0 indicates that the full test set of 411 proteins was used in the analysis, and a cutoff of 0.5 indicates that only proteins with above 50% unordered content were used in the analysis. To see this figure in color, go online.

demonstrated in our equilibrium peptide simulations, which were performed for all mutant variations and at different temperatures (Fig. 3). The effect of temperature on the secondary structures of each peptide appears to be minimal. In each simulation, the helical conformation is overexpressed regardless of temperature or even mutation. In all three peptide runs at each temperature, the same structural trend appears: helix, turn, and unordered structure components are similarly distributed. Compared with the experimental CD results (Fig. 1), we expect the emergence of a dominant structure in AAA that does not appear in RRK or RAK. Because the trajectory data do not display this trend, the force field we used is assumed to contain conformational bias despite previous efforts to improve accuracy (27).

Newer force fields for MD simulations that are designed for IDPs and folded proteins are available (65,66). However, choosing the best model for our specific system was not a simple task. In addition, variations in the water model heavily affect the outcome of IDP simulations (67,68). There is a limitation to IDP force field development because of the lack of experimental data detailing the conformational ensemble of IDPs. Common methods for experimentally refining force fields such as small-angle X-ray scattering (SAXS), fluorescence resonance energy transfer (FRET), and NMR are only able to produce an average of the conformational ensemble (34,69) and do not necessarily contain the observables needed to describe IDPs in silico. We elected to sample a larger set of data by implementing an implicit solvent model instead of focusing our efforts on finding the best MD parameterization. By combining simulation and experimental results, we are able to reveal and partially resolve the shortcomings in each method (70).

Conformations of unbound CaMKII peptide may be important to binding with CaM

The experimental study of the CaM-CaMKII binding kinetics between CaM and the CaMKII peptides illustrate an ~6-fold increase in the association rate of RRK compared with AAA (41) in 150 mM ionic solution. This ionic strength effectively screens the electrostatic potential by a Debye length of 7.8 Å^{-1}. This screening effect can decrease the electrostatic rate enhancement for diffusion-limited binding kinetics (71,72) of CaM and the CaMKII peptides; however, the electrostatic potential is not completely screened over localized peptide regions. Comparison of the kinetic results to the conformational analysis in this study resolves a finite set of possible binding mechanisms between CaM and the CaMKII peptides. We initially assumed that AAA would have a higher affinity for CaM because of the residual helical propensity induced by the alanine residues because these peptides are known to adopt a helical conformation when they bind to CaM. It has been hypothesized that the presence of a residual structure that resembles the bound state increases the rate of association (73,74). Because the stopped-flow experimental results (decreased on-rate for AAA relative to RRK (41)) disproved this hypothesis, we turned to our CD analysis, which has shown to offer a diverse range of secondary structures for other CaM-binding target peptides (75), for additional potential mechanisms.

The CD measurements indicate a distinct difference in the ensemble of RRK and AAA secondary structures. Our CD deconvolution results indicate that the secondary structure formed through each mutation is actually in the form of a hairpin structure. The apparent lack of helical structure in the peptide ensemble implies that the hypothesis that increased kinetics and peptide residual structure are positively correlated (76–78) is not applicable in modeling the CaM-CaMKII peptide binding. Moreover, a larger energy gap between the bound and unbound states may exist because of the presence of the stable hairpin structure in AAA (79–81). For the mutual and induced conformational fit mechanism (82) to take place, the peptide must transition from the hairpin structure to the extended state to form productive and stable contacts with CaM. Our findings suggest that a significant conformational change must occur for the AAA peptide, reversing the hairpin structure to allow formation of the helical conformation upon formation of the CaM-bound complex (83). This provides a plausible

mechanistic explanation for the differences in association rates (41) and emphasizes that conformational frustration can be an important step in regulating the kinetics of protein-protein interactions.

CONCLUSIONS

The importance of IDPs in biological function has become readily apparent in recent years. A major challenge in IDP modeling stems from experimental sampling of the structure ensemble. Popular methods such as NMR spectroscopy offer higher resolution but are still limited in IDP ensemble determination. To overcome difficulties pertaining to experimental ensemble construction of IDPs, combined theoretical approaches are often used. CD spectroscopy does not offer high-resolution structure determination; however, this drawback appears to be inconsequential for IDPs because MD simulation can be used to perturb the averaged structure to generate the IDP ensemble. In this study, we have used a combination of techniques to bridge the experimental data with theoretical data to generate a detailed picture of our CaMKII peptides despite the inherent inaccuracy of the MD simulation. Our resulting ensemble approximations illustrate how the residual secondary structure of the CaMKII peptides changes because of charged residue mutation. Our findings suggest that the AAA ensemble becomes stabilized through the formation of the hairpin secondary structure, which may explain the binding phenomenon observed in previous studies (41). In addition to the free-peptide ensemble, the observed structure shift may play a significant role in complex stability postbinding because of the formation (or lack thereof) of "fuzzy structures" (84,85).

SUPPORTING MATERIAL

Supporting Material can be found online at https://doi.org/10.1016/j.bpj.2020.02.015.

AUTHOR CONTRIBUTIONS

M.N.W. and M.S.C. designed research. J.C.E. and P.Z. performed research. J.C.E., P.Z., and N.C.J. analyzed data. J.C.E., P.Z., M.S.C., and M.N.W. wrote the article.

ACKNOWLEDGMENTS

M.S.C. and M.N.W. are grateful for the support from the National Institute of Health (2R01GM097553). J.C.E. is supported by a training fellowship from the Gulf Coast Consortia to the Houston Area Molecular Biophysics Program (National Institute of General Medical Sciences grant T32GM008280). The computational resource is uHPC at the University of Houston supported by the National Science Foundation (OAC: 1531814). M.S.C. acknowledges support from the Moores Professorship. M.N.W. also acknowledges support from an endowment from the William Wheless III Professorship.

REFERENCES

1. Eliezer, D. 2009. Biophysical characterization of intrinsically disordered proteins. *Curr. Opin. Struct. Biol.* 19:23–30.
2. Dunker, A. K., J. D. Lawson, …, Z. Obradovic. 2001. Intrinsically disordered protein. *J. Mol. Graph. Model.* 19:26–59.
3. Tran, H. T., A. Mao, and R. V. Pappu. 2008. Role of backbone-solvent interactions in determining conformational equilibria of intrinsically disordered proteins. *J. Am. Chem. Soc.* 130:7380–7392.
4. Uversky, V. N., C. J. Oldfield, and A. K. Dunker. 2005. Showing your ID: intrinsic disorder as an ID for recognition, regulation and cell signaling. *J. Mol. Recognit.* 18:343–384.
5. Pauwels, K., P. Lebrun, and P. Tompa. 2017. To be disordered or not to be disordered: is that still a question for proteins in the cell? *Cell. Mol. Life Sci.* 74:3185–3204.
6. Borgia, A., M. B. Borgia, …, B. Schuler. 2018. Extreme disorder in an ultrahigh-affinity protein complex. *Nature.* 555:61–66.
7. Wright, P. E., and H. J. Dyson. 2015. Intrinsically disordered proteins in cellular signalling and regulation. *Nat. Rev. Mol. Cell Biol.* 16:18–29.
8. Iakoucheva, L. M., C. J. Brown, …, A. K. Dunker. 2002. Intrinsic disorder in cell-signaling and cancer-associated proteins. *J. Mol. Biol.* 323:573–584.
9. Grundke-Iqbal, I., K. Iqbal, …, L. I. Binder. 1986. Abnormal phosphorylation of the microtubule-associated protein tau (tau) in Alzheimer cytoskeletal pathology. *Proc. Natl. Acad. Sci. USA.* 83:4913–4917.
10. Mudher, A., and S. Lovestone. 2002. Alzheimer's disease-do tauists and baptists finally shake hands? *Trends Neurosci.* 25:22–26.
11. Levine, Z. A., L. Larini, …, J. E. Shea. 2015. Regulation and aggregation of intrinsically disordered peptides. *Proc. Natl. Acad. Sci. USA.* 112:2758–2763.
12. Wright, P. E., and H. J. Dyson. 2009. Linking folding and binding. *Curr. Opin. Struct. Biol.* 19:31–38.
13. Fuxreiter, M. 2019. Fold or not to fold upon binding - does it really matter? *Curr. Opin. Struct. Biol.* 54:19–25.
14. Ball, K. A., A. H. Phillips, …, T. Head-Gordon. 2011. Homogeneous and heterogeneous tertiary structure ensembles of amyloid-β peptides. *Biochemistry.* 50:7612–7628.
15. Fisher, C. K., and C. M. Stultz. 2011. Constructing ensembles for intrinsically disordered proteins. *Curr. Opin. Struct. Biol.* 21:426–431.
16. Gong, H., S. Zhang, …, J. Zeng. 2016. Constructing structure ensembles of intrinsically disordered proteins from chemical shift data. *J. Comput. Biol.* 23:300–310.
17. Lindorff-Larsen, K., S. Kristjansdottir, …, M. Vendruscolo. 2004. Determination of an ensemble of structures representing the denatured state of the bovine acyl-coenzyme a binding protein. *J. Am. Chem. Soc.* 126:3291–3299.
18. Dyson, H. J., and P. E. Wright. 2005. Intrinsically unstructured proteins and their functions. *Nat. Rev. Mol. Cell Biol.* 6:197–208.
19. Collins, A. P., and P. C. Anderson. 2018. Complete coupled binding-folding pathway of the intrinsically disordered transcription factor protein brinker revealed by molecular dynamics simulations and Markov state modeling. *Biochemistry.* 57:4404–4420.
20. Van Roey, K., T. J. Gibson, and N. E. Davey. 2012. Motif switches: decision-making in cell regulation. *Curr. Opin. Struct. Biol.* 22:378–385.
21. Borg, M., T. Mittag, …, H. S. Chan. 2007. Polyelectrostatic interactions of disordered ligands suggest a physical basis for ultrasensitivity. *Proc. Natl. Acad. Sci. USA.* 104:9650–9655.
22. Lee, C. W., J. C. Ferreon, …, P. E. Wright. 2010. Graded enhancement of p53 binding to CREB-binding protein (CBP) by multisite phosphorylation. *Proc. Natl. Acad. Sci. USA.* 107:19290–19295.
23. Gsponer, J., and M. M. Babu. 2009. The rules of disorder or why disorder rules. *Prog. Biophys. Mol. Biol.* 99:94–103.
24. Brutscher, B., I. C. Felli, …, Z. Sólyom. 2015. NMR methods for the study of instrinsically disordered proteins structure, dynamics, and

interactions: general overview and practical guidelines. *In* Intrinsically Disordered Proteins Studied by NMR Spectroscopy. I. C. Felli and R. Pierattelli, eds. Springer International Publishing, pp. 49–122.

25. Best, R. B., N. V. Buchete, and G. Hummer. 2008. Are current molecular dynamics force fields too helical? *Biophys. J.* 95:L07–L09.
26. Hornak, V., R. Abel, …, C. Simmerling. 2006. Comparison of multiple Amber force fields and development of improved protein backbone parameters. *Proteins.* 65:712–725.
27. Lindorff-Larsen, K., S. Piana, …, D. E. Shaw. 2010. Improved side-chain torsion potentials for the Amber ff99SB protein force field. *Proteins.* 78:1950–1958.
28. Ota, M., R. Koike, …, S. Fukuchi. 2013. An assignment of intrinsically disordered regions of proteins based on NMR structures. *J. Struct. Biol.* 181:29–36.
29. Brookes, D. H., and T. Head-Gordon. 2016. Experimental inferential structure determination of ensembles for intrinsically disordered proteins. *J. Am. Chem. Soc.* 138:4530–4538.
30. Navarro-Retamal, C., A. Bremer, …, A. Thalhammer. 2016. Molecular dynamics simulations and CD spectroscopy reveal hydration-induced unfolding of the intrinsically disordered LEA proteins COR15A and COR15B from *Arabidopsis thaliana*. *Phys. Chem. Chem. Phys.* 18:25806–25816.
31. Xu, X.-P., and D. A. Case. 2001. Automated prediction of 15N, 13Calpha, 13Cbeta and 13C′ chemical shifts in proteins using a density functional database. *J. Biomol. NMR.* 21:321–333.
32. Lincoff, J., S. Sasmal, and T. Head-Gordon. 2019. The combined force field-sampling problem in simulations of disordered amyloid-β peptides. *J. Chem. Phys.* 150:104108.
33. Cavalli, A., X. Salvatella, …, M. Vendruscolo. 2007. Protein structure determination from NMR chemical shifts. *Proc. Natl. Acad. Sci. USA.* 104:9615–9620.
34. Jensen, M. R., M. Zweckstetter, …, M. Blackledge. 2014. Exploring free-energy landscapes of intrinsically disordered proteins at atomic resolution using NMR spectroscopy. *Chem. Rev.* 114:6632–6660.
35. Wang, W., W. Ye, …, H. F. Chen. 2014. New force field on modeling intrinsically disordered proteins. *Chem. Biol. Drug Des.* 84:253–269.
36. Kumagai, P. S., R. DeMarco, and J. L. S. Lopes. 2017. Advantages of synchrotron radiation circular dichroism spectroscopy to study intrinsically disordered proteins. *Eur. Biophys. J.* 46:599–606.
37. Whitmore, L., and B. A. Wallace. 2008. Protein secondary structure analyses from circular dichroism spectroscopy: methods and reference databases. *Biopolymers.* 89:392–400.
38. Sreerama, N., and R. W. Woody. 2000. Estimation of protein secondary structure from circular dichroism spectra: comparison of CONTIN, SELCON, and CDSSTR methods with an expanded reference set. *Anal. Biochem.* 287:252–260.
39. Sreerama, N., S. Y. Venyaminov, and R. W. Woody. 2000. Estimation of protein secondary structure from circular dichroism spectra: inclusion of denatured proteins with native proteins in the analysis. *Anal. Biochem.* 287:243–251.
40. Provencher, S. W., and J. Glöckner. 1981. Estimation of globular protein secondary structure from circular dichroism. *Biochemistry.* 20:33–37.
41. Waxham, M. N., A. L. Tsai, and J. A. Putkey. 1998. A mechanism for calmodulin (CaM) trapping by CaM-kinase II defined by a family of CaM-binding peptides. *J. Biol. Chem.* 273:17579–17584.
42. Sreerama, N., and R. W. Woody. 2004. On the analysis of membrane protein circular dichroism spectra. *Protein Sci.* 13:100–112.
43. Whitmore, L., B. Woollett, …, B. A. Wallace. 2011. PCDDB: the Protein Circular Dichroism Data Bank, a repository for circular dichroism spectral and metadata. *Nucleic Acids Res.* 39:D480–D486.
44. Case, D. A., V. Babin, …, R. M. Betz. 2014. AMBER 14. University of California, San Francisco, CA.
45. Srinivasan, J., M. W. Trevathan, …, D. A. Case. 1999. Application of a pairwise generalized Born model to proteins and nucleic acids: inclusion of salt effects. *Theor. Chem. Acc.* 101:426–434.
46. Onufriev, A., D. Bashford, and D. A. Case. 2004. Exploring protein native states and large-scale conformational changes with a modified generalized born model. *Proteins.* 55:383–394.
47. Nguyen, H., D. R. Roe, and C. Simmerling. 2013. Improved generalized born solvent model parameters for protein simulations. *J. Chem. Theory Comput.* 9:2020–2034.
48. Ryckaert, J.-P., G. Ciccotti, and H. J. C. Berendsen. 1977. Numerical integration of the cartesian equations of motion of a system with constraints: molecular dynamics of n-alkanes. *J. Comput. Phys.* 23:327–341.
49. Sindhikara, D. J., S. Kim, …, A. E. Roitberg. 2009. Bad seeds sprout perilous dynamics: stochastic thermostat induced trajectory synchronization in biomolecules. *J. Chem. Theory Comput.* 5:1624–1631.
50. Eguchi, S., and J. Copas. 2006. Interpreting Kullback–Leibler divergence with the Neyman–Pearson lemma. *J. Multivariate Anal.* 97:2034–2040.
51. Kullback, S., and R. A. Leibler. 1951. On information and sufficiency. *Ann. Math. Stat.* 22:79–86.
52. Roe, D. R., and T. E. Cheatham, III. 2013. PTRAJ and CPPTRAJ: software for processing and analysis of molecular dynamics trajectory data. *J. Chem. Theory Comput.* 9:3084–3095.
53. Kabsch, W., and C. Sander. 1983. Dictionary of protein secondary structure: pattern recognition of hydrogen-bonded and geometrical features. *Biopolymers.* 22:2577–2637.
54. Greenfield, N. J. 2006. Using circular dichroism spectra to estimate protein secondary structure. *Nat. Protoc.* 1:2876–2890.
55. Pace, C. N., and J. M. Scholtz. 1998. A helix propensity scale based on experimental studies of peptides and proteins. *Biophys. J.* 75:422–427.
56. Wiedemann, C., P. Bellstedt, and M. Görlach. 2013. CAPITO–a web server-based analysis and plotting tool for circular dichroism data. *Bioinformatics.* 29:1750–1757.
57. Micsonai, A., F. Wien, …, J. Kardos. 2018. BeStSel: a web server for accurate protein secondary structure prediction and fold recognition from the circular dichroism spectra. *Nucleic Acids Res.* 46:W315–W322.
58. Ezerski, J. C., and M. S. Cheung. 2018. CATS: a tool for clustering the ensemble of intrinsically disordered peptides on a flat energy landscape. *J. Phys. Chem. B.* 122:11807–11816.
59. Das, R. K., and R. V. Pappu. 2013. Conformations of intrinsically disordered proteins are influenced by linear sequence distributions of oppositely charged residues. *Proc. Natl. Acad. Sci. USA.* 110:13392–13397.
60. Mao, A. H., S. L. Crick, …, R. V. Pappu. 2010. Net charge per residue modulates conformational ensembles of intrinsically disordered proteins. *Proc. Natl. Acad. Sci. USA.* 107:8183–8188.
61. Pace, C. N., R. W. Alston, and K. L. Shaw. 2000. Charge-charge interactions influence the denatured state ensemble and contribute to protein stability. *Protein Sci.* 9:1395–1398.
62. Holehouse, A. S., J. Ahad, …, R. V. Pappu. 2015. CIDER: classification of intrinsically disordered ensemble regions. *Biophys. J.* 108:228a.
63. Huang, J., and A. D. MacKerell, Jr. 2013. CHARMM36 all-atom additive protein force field: validation based on comparison to NMR data. *J. Comput. Chem.* 34:2135–2145.
64. Lange, O. F., D. van der Spoel, and B. L. de Groot. 2010. Scrutinizing molecular mechanics force fields on the submicrosecond timescale with NMR data. *Biophys. J.* 99:647–655.
65. Robustelli, P., S. Piana, and D. E. Shaw. 2018. Developing a molecular dynamics force field for both folded and disordered protein states. *Proc. Natl. Acad. Sci. USA.* 115:E4758–E4766.
66. Huang, J., S. Rauscher, …, A. D. MacKerell, Jr. 2017. CHARMM36m: an improved force field for folded and intrinsically disordered proteins. *Nat. Methods.* 14:71–73.
67. Best, R. B., and J. Mittal. 2010. Protein simulations with an optimized water model: cooperative helix formation and temperature-induced unfolded state collapse. *J. Phys. Chem. B.* 114:14916–14923.

68. Boonstra, S., P. R. Onck, and E. van der Giessen. 2016. CHARMM TIP3P water model suppresses peptide folding by solvating the unfolded state. *J. Phys. Chem. B.* 120:3692–3698.

69. Brucale, M., B. Schuler, and B. Samorì. 2014. Single-molecule studies of intrinsically disordered proteins. *Chem. Rev.* 114:3281–3317.

70. Fisher, C. K., A. Huang, and C. M. Stultz. 2010. Modeling intrinsically disordered proteins with bayesian statistics. *J. Am. Chem. Soc.* 132:14919–14927.

71. Vijayakumar, M., K. Y. Wong, …, H. X. Zhou. 1998. Electrostatic enhancement of diffusion-controlled protein-protein association: comparison of theory and experiment on barnase and barstar. *J. Mol. Biol.* 278:1015–1024.

72. Alsallaq, R., and H. X. Zhou. 2008. Electrostatic rate enhancement and transient complex of protein-protein association. *Proteins.* 71:320–335.

73. Radivojac, P., S. Vucetic, …, A. K. Dunker. 2006. Calmodulin signaling: analysis and prediction of a disorder-dependent molecular recognition. *Proteins.* 63:398–410.

74. Mohan, A., C. J. Oldfield, …, V. N. Uversky. 2006. Analysis of molecular recognition features (MoRFs). *J. Mol. Biol.* 362:1043–1059.

75. Dunlap, T. B., J. M. Kirk, …, T. P. Creamer. 2013. Thermodynamics of binding by calmodulin correlates with target peptide α-helical propensity. *Proteins.* 81:607–612.

76. Csermely, P., R. Palotai, and R. Nussinov. 2010. Induced fit, conformational selection and independent dynamic segments: an extended view of binding events. *Trends Biochem. Sci.* 35:539–546.

77. Arai, M., K. Sugase, …, P. E. Wright. 2015. Conformational propensities of intrinsically disordered proteins influence the mechanism of binding and folding. *Proc. Natl. Acad. Sci. USA.* 112:9614–9619.

78. Iešmantavičius, V., J. Dogan, …, M. Kjaergaard. 2014. Helical propensity in an intrinsically disordered protein accelerates ligand binding. *Angew. Chem. Int.Engl.* 53:1548–1551.

79. Weinkam, P., E. V. Pletneva, …, P. G. Wolynes. 2009. Electrostatic effects on funneled landscapes and structural diversity in denatured protein ensembles. *Proc. Natl. Acad. Sci. USA.* 106:1796–1801.

80. Higo, J., N. Ito, …, H. Nakamura. 2001. Energy landscape of a peptide consisting of α-helix, 3(10)-helix, β-turn, β-hairpin, and other disordered conformations. *Protein Sci.* 10:1160–1171.

81. Higo, J., Y. Nishimura, and H. Nakamura. 2011. A free-energy landscape for coupled folding and binding of an intrinsically disordered protein in explicit solvent from detailed all-atom computations. *J. Am. Chem. Soc.* 133:10448–10458.

82. Wang, Q., P. Zhang, …, M. S. Cheung. 2013. Protein recognition and selection through conformational and mutually induced fit. *Proc. Natl. Acad. Sci. USA.* 110:20545–20550.

83. Meador, W. E., A. R. Means, and F. A. Quiocho. 1993. Modulation of calmodulin plasticity in molecular recognition on the basis of x-ray structures. *Science.* 262:1718–1721.

84. Tompa, P., and M. Fuxreiter. 2008. Fuzzy complexes: polymorphism and structural disorder in protein-protein interactions. *Trends Biochem. Sci.* 33:2–8.

85. Wu, H., and M. Fuxreiter. 2016. The structure and dynamics of higher-order assemblies: amyloids, signalosomes, and granules. *Cell.* 165:1055–1066.

86. Micsonai, A., F. Wien, …, J. Kardos. 2015. Accurate secondary structure prediction and fold recognition for circular dichroism spectroscopy. *Proc. Natl. Acad. Sci. USA.* 112:E3095–E3103.

Biophysical Journal, Volume *118*

Supplemental Information

Molecular Dynamics Ensemble Refinement of Intrinsically Disordered Peptides According to Deconvoluted Spectra from Circular Dichroism

Jacob C. Ezerski, Pengzhi Zhang, Nathaniel C. Jennings, M. Neal Waxham, and Margaret S. Cheung

SUPPLEMENTARY INFORMATION

Tables:

Table S1 The fractions of secondary structures from CAPITO deconvolution of the CD spectra for the CaMKII peptides. Normalized root mean squared deviation (NRMSD) is a dimensionless parameter to assess the goodness of the fitting as defined by Wiedemann et al. [1]

	helix	β-sheet	irregular	NRMSD
RRK	0.32	0.01	0.68	1.55
RAK	0.21	0.01	0.78	0.73
AAA	0.53	0.06	0.41	0.89

Table S2 Fractional secondary structures derived from CD deconvolution using the non-negative least squares fitting method in conjunction with the SDP48, SP37A, SMP56 and SP43 reference data sets. SDP48 is the data set including denatured proteins. Others include the data set of globular proteins.

Data set	Peptide	Helix	Strand	Turn	Unordered	RMSD (Δϵ)
SDP48	RRK	0.04	0.15	0.09	0.72	0.26
	RAK	0.04	0.21	0.13	0.62	0.23
	AAA	0.04	0.34	0.17	0.46	0.36
SP37A	RRK	0.11	0.35	0.20	0.34	2.14
	RAK	0.11	0.35	0.20	0.34	0.97
	AAA	0.08	0.39	0.22	0.32	0.65
SMP56	RRK	0.11	0.35	0.20	0.34	2.14
	RAK	0.11	0.35	0.20	0.34	0.97
	AAA	0.09	0.39	0.20	0.32	0.64
SP43	RRK	0.11	0.35	0.20	0.34	2.14
	RAK	0.11	0.35	0.20	0.34	0.97
	AAA	0.09	0.39	0.20	0.32	0.64

Table S3 Comparison of the structure ensembles of RRK, RAK, and AAA peptides. Root mean square deviation (RMSD) from the averaged structure is calculated for each peptide ensemble based on backbone heavy atoms. The average ($\overline{RMSD}$), standard deviation (σ_{RMSD}), the minimum ($RMSD_{min}$), and maximum ($RMSD_{max}$) values of the RMSD are provided.

	$\overline{RMSD}$ (Å)	σ_{RMSD} (Å)	$RMSD_{min}$ (Å)	$RMSD_{max}$ (Å)	number of structures
RRK	4.6	1.4	2.3	12.5	11002
RAK	4.3	1.7	2.1	12.4	2410
AAA	4.7	1.0	3.5	10.5	130

Table S4 Sequence analysis of RRK, RAK and AAA using CIDER.

peptide	κ	FCR	NCPR	hydropathy	disorder promoting
RRK	0.55	0.25	0.25	4.26	0.6
RAK	0.383	0.2	0.2	4.575	0.6
AAA	0.313	0.1	0.1	5.175	0.6

κ represents an order parameter indicating charge segregation within the peptide; FCR is the fraction of charged residues; NCPR is the linear net charge per residue; hydropathy indicates hydrophobicity and ranges from 0 to 9; the fraction of disorder-promoting residues defined by Dunker [2] is provided.

Table S5 Kullback-Leibler divergence between distributions of total potential energy of the peptides over accumulated simulation time.

Peptide	RRK			RAK			AAA		
Temp (K)	277	285	293	277	285	293	277	285	293
1.2 μs	0.10	0.06	0.02	0.03	0.02	0.05	0.08	0.02	0.02
1.8 μs	0.10	0.01	0.01	0.10	0.02	0.01	0.07	0.01	0.01
2.4 μs	0.04	0.01	0.01	0.06	0.01	0.01	0.04	0.01	0.01
3.0 μs	0.01	N/A		0.03	N/A		0.02	N/A	
3.6 μs	0.01			0.01			0.02		
4.2 μs	0.01			0.01			0.02		
4.8 μs	0.01			0.01			0.01		

Table S6 Details pertaining to the Hieragglo clusters produced by CPPTRAJ are shown for RRK, RAK, and AAA.

peptide	# Cluster	# of frames	fraction	AvgDist (A)	Stdev (A)	Centroid	AvgCDist (A)
RRK	0	7914	0.719	4.5	1.2	9925	8.2
	1	2292	0.208	4.0	1.3	4519	7.7
	2	171	0.016	4.8	1.3	5228	7.1
	3	148	0.013	4.6	1.3	1796	7.0
	4	135	0.012	5.2	1.2	2519	6.9
	5	105	0.01	4.2	1.7	90	7.5
	6	98	0.009	4.7	0.9	16	8.3
	7	87	0.008	5.2	1.0	450	6.9
	8	47	0.004	4.8	1.2	2366	7.4
	9	5	0.0	4.3	1.3	10996	7.2
RAK	0	1883	0.781	4.5	1.6	2042	7.7
	1	197	0.082	4.9	1.2	284	7.1
	2	109	0.045	4.7	1.1	186	8.3
	3	104	0.043	4.4	1.8	1047	8.1
	4	62	0.026	4.1	1.5	1421	7.2
	5	25	0.01	4.1	1.4	608	7.0
	6	14	0.006	4.2	1.3	175	7.4
	7	6	0.002	4.5	0.8	166	7.0
	8	5	0.002	4.7	1.3	233	6.9
	9	5	0.002	3.5	1.0	1532	7.3
AAA	0	59	0.454	2.1	0.8	70	6.6
	1	27	0.208	2.5	0.9	19	6.2
	2	25	0.192	2.4	0.9	41	5.5
	3	9	0.069	1.5	0.5	52	6.0
	4	3	0.023	1.1	0.1	28	5.9
	5	3	0.023	1.9	0.3	128	5.5
	6	1	0.008	0.0	0.0	124	6.9
	7	1	0.008	0	0	125	6.5
	8	1	0.008	0	0	126	5.4
	9	1	0.008	0	0	130	6.9

Table S7 Performance indices for varying subsets of unordered content indicated by a minimum structure content cutoff value. The number of proteins satisfying the cutoff criteria is given by column N. Cells highlighted in red indicate CDPro algorithms are the highest performing methods and cells highlighted in green indicate NN-LSQ is the best performing method.

cut(unorder)	N	Method	σ(H)	r(H)	σ(β)	r(β)	σ(T)	r(T)	σ(U)	r(U)	σ	r
0	411	NN-LSQ	0.1223	0.6953	0.1064	0.6254	0.0623	0.3225	0.1064	0.1601	0.1018	0.6006
		SELCON3	0.1156	0.7023	0.0902	0.6455	0.0537	0.294	0.0863	0.2236	0.0892	0.7147
		CDSSTR	0.1196	0.7255	0.0855	0.7117	0.0604	0.2689	0.0963	0.2519	0.0929	0.7173
		CONTIN/LL	0.1073	0.7632	0.0881	0.698	0.0557	0.2396	0.0921	0.244	0.0878	0.7286
0.1	409	NN-LSQ	0.1213	0.6897	0.1062	0.6239	0.0623	0.3189	0.106	0.1505	0.1014	0.5962
		SELCON3	0.1147	0.696	0.0904	0.642	0.0518	0.31	0.0856	0.2205	0.0885	0.7119
		CDSSTR	0.119	0.7207	0.0856	0.7092	0.06	0.2692	0.0963	0.2318	0.0927	0.7122
		CONTIN/LL	0.1064	0.7595	0.0883	0.6952	0.0552	0.2369	0.0919	0.2276	0.0874	0.7243
0.2	382	NN-LSQ	0.1135	0.656	0.1024	0.6206	0.0631	0.2836	0.1047	0.0967	0.0979	0.561
		SELCON3	0.1137	0.599	0.0868	0.6354	0.0507	0.1805	0.0866	0.0808	0.0874	0.6555
		CDSSTR	0.1158	0.6295	0.0826	0.7097	0.0582	0.1305	0.0978	0.0368	0.0911	0.6511
		CONTIN/LL	0.1076	0.6698	0.0881	0.6615	0.0531	0.1002	0.0934	0.0859	0.0879	0.6582
0.3	31	NN-LSQ	0.101	0.8088	0.0949	0.7596	0.0801	-0.0088	0.2653	-0.1158	0.1549	0.5588
		SELCON3	0.1666	0.6069	0.1163	0.6399	0.0565	0.4048	0.2631	-0.2124	0.1686	0.4654
		CDSSTR	0.1686	0.7112	0.0964	0.7513	0.0647	0.3546	0.2836	-0.1656	0.1749	0.4766
		CONTIN/LL	0.1274	0.7705	0.097	0.7864	0.0872	-0.0871	0.2837	-0.2301	0.1686	0.5025
0.4	10	NN-LSQ	0.079	0.8621	0.1032	0.6008	0.1256	-0.6799	0.4481	-0.5362	0.2416	0.4066
		SELCON3	0.2378	0.2569	0.155	0.0611	0.0732	0.2234	0.4434	-0.4839	0.2658	0.203
		CDSSTR	0.2213	0.4954	0.1025	0.5432	0.086	-0.0818	0.4664	-0.4174	0.2667	0.2552
		CONTIN/LL	0.1525	0.6468	0.1197	0.516	0.136	-0.7972	0.4739	-0.5733	0.2649	0.2813
0.5	3	NN-LSQ	0.0938	0.9636	0.0984	-0.9721	0.0536	0.9672	0.7507	0.9204	0.3824	0.4693
		SELCON3	0.159	0.6424	0.0957	-0.7226	0.0593	0.4159	0.7767	0.5898	0.4004	0.236
		CDSSTR	0.2305	0.6337	0.0797	-0.6898	0.0953	0.5752	0.7986	0.6375	0.4202	0.152
		CONTIN/LL	0.1913	0.618	0.0945	-0.7393	0.0615	0.5737	0.7984	0.4635	0.4144	0.1531

Table S8 Performance indices for varying subsets of beta content indicated by a minimum structure content cutoff value. The number of proteins satisfying the cutoff criteria is given by column N. Cells highlighted in red indicate CDPro algorithms are the highest performing methods and cells highlighted in green indicate NN-LSQ is the best performing method.

cut(Strand)	N	Method	σ(H)	r(H)	σ(β)	r(β)	σ(T)	r(T)	σ(U)	r(U)	σ	r
0	411	NN-LSQ	0.1223	0.6953	0.1064	0.6254	0.0623	0.3225	0.1064	0.1601	0.1018	0.6006
		SELCON3	0.1156	0.7023	0.0902	0.6455	0.0537	0.294	0.0863	0.2236	0.0892	0.7147
		CDSSTR	0.1196	0.7255	0.0855	0.7117	0.0604	0.2689	0.0963	0.2519	0.0929	0.7173
		CONTIN/LL	0.1073	0.7632	0.0881	0.698	0.0557	0.2396	0.0921	0.244	0.0878	0.7286
0.1	362	NN-LSQ	0.113	0.6376	0.106	0.5707	0.0626	-0.069	0.1066	0.0882	0.0991	0.5114
		SELCON3	0.1188	0.5111	0.0936	0.5689	0.0467	0.0064	0.0869	0.1162	0.0903	0.5952
		CDSSTR	0.1195	0.5548	0.0889	0.6485	0.0514	-0.0428	0.0948	0.1028	0.0919	0.6067
		CONTIN/LL	0.1095	0.6211	0.0921	0.6312	0.048	-0.0702	0.0925	0.1473	0.0885	0.6195
0.2	58	NN-LSQ	0.0878	0.6399	0.1151	0.6107	0.0571	0.0995	0.0892	0.365	0.0897	0.7253
		SELCON3	0.1462	0.3413	0.1412	0.5324	0.0669	0.0429	0.0938	0.3107	0.1168	0.5429
		CDSSTR	0.1315	0.4752	0.1201	0.5896	0.0557	0.2898	0.0862	0.4486	0.1028	0.6633
		CONTIN/LL	0.1032	0.556	0.1169	0.6527	0.0742	-0.2346	0.1062	0.4373	0.1014	0.6769
0.3	42	NN-LSQ	0.0922	0.4555	0.1288	0.5183	0.0592	0.0527	0.087	0.4293	0.0951	0.7644
		SELCON3	0.1648	-0.0426	0.1584	0.282	0.0578	0.2556	0.0977	0.3222	0.1276	0.5595
		CDSSTR	0.1445	0.0452	0.1285	0.4957	0.0595	0.2715	0.0921	0.4674	0.1112	0.6826
		CONTIN/LL	0.1125	0.0946	0.1265	0.5406	0.079	-0.216	0.1096	0.4625	0.1083	0.7024
0.4	23	NN-LSQ	0.0782	0.4687	0.1519	0.1128	0.0662	-0.3074	0.0916	0.1556	0.1024	0.8132
		SELCON3	0.1897	0.0128	0.2007	0.2033	0.0562	0.4744	0.0973	0.0146	0.149	0.536
		CDSSTR	0.1582	0.0748	0.1526	0.1849	0.0595	0.4935	0.0903	-0.1077	0.1225	0.7038
		CONTIN/LL	0.108	0.0064	0.1489	0.1324	0.0937	-0.3867	0.1228	-0.0335	0.1201	0.7204
0.5	7	NN-LSQ	0.0925	0.0935	0.2384	-0.1961	0.0716	0.6353	0.1223	0.461	0.1462	0.7712
		SELCON3	0.206	0.7873	0.265	0.3653	0.0862	0.4311	0.079	0.1478	0.1777	0.5785
		CDSSTR	0.1785	0.8185	0.232	0.3343	0.0884	0.3004	0.0614	0.1436	0.1559	0.7061
		CONTIN/LL	0.0956	0.883	0.1888	0.5545	0.1081	-0.4405	0.0976	0.3249	0.1285	0.8417

Table S9 Performance indices for varying subsets of helix content indicated by a minimum structure content cutoff value. The number of proteins satisfying the cutoff criteria is given by column N. Cells highlighted in red indicate CDPro algorithms are the best performing methods.

cut(Helix)	N	Method	σ(H)	r(H)	σ(β)	r(β)	σ(T)	r(T)	σ(U)	r(U)	σ	r
0	411	NN-LSQ	0.1223	0.6953	0.1064	0.6254	0.0623	0.3225	0.1064	0.1601	0.1018	0.6006
		SELCON3	0.1156	0.7023	0.0902	0.6455	0.0537	0.294	0.0863	0.2236	0.0892	0.7147
		CDSSTR	0.1196	0.7255	0.0855	0.7117	0.0604	0.2689	0.0963	0.2519	0.0929	0.7173
		CONTIN/LL	0.1073	0.7632	0.0881	0.698	0.0557	0.2396	0.0921	0.244	0.0878	0.7286
0.3	349	NN-LSQ	0.1284	0.5175	0.1051	0.4096	0.06	0.4654	0.1059	0.0661	0.1029	0.5835
		SELCON3	0.1095	0.7455	0.079	0.4808	0.0492	0.3902	0.0851	0.1833	0.0835	0.749
		CDSSTR	0.1178	0.7344	0.0789	0.5047	0.0593	0.3481	0.0965	0.2099	0.0907	0.7298
		CONTIN/LL	0.1094	0.7416	0.0828	0.4907	0.0492	0.4211	0.0874	0.1843	0.085	0.7418
0.4	60	NN-LSQ	0.1754	0.4266	0.1072	0.382	0.0561	0.8437	0.1026	0.2175	0.1183	0.7966
		SELCON3	0.1019	0.8184	0.0626	0.5809	0.0841	0.6933	0.0788	0.3919	0.083	0.9123
		CDSSTR	0.1307	0.7961	0.0582	0.6292	0.0975	0.7272	0.0991	0.4106	0.0997	0.9033
		CONTIN/LL	0.1024	0.7996	0.0592	0.5723	0.0881	0.7568	0.0855	0.465	0.0852	0.9111
0.5	29	NN-LSQ	0.2175	0.0229	0.1313	0.0433	0.0431	0.3811	0.1121	0.1085	0.1405	0.8237
		SELCON3	0.0956	0.6264	0.0473	0.4151	0.0772	-0.1275	0.0753	0.3274	0.0758	0.9557
		CDSSTR	0.1428	0.5189	0.0556	0.3421	0.0786	0.1437	0.0994	0.5186	0.0994	0.9474
		CONTIN/LL	0.0969	0.5642	0.0453	0.4476	0.0676	0.1969	0.0663	0.5269	0.0714	0.9611
0.6	18	NN-LSQ	0.251	0.125	0.1415	0.5355	0.042	0.2448	0.1176	0.213	0.157	0.8304
		SELCON3	0.1089	0.3133	0.0378	0.1845	0.0946	-0.5374	0.0765	0.1061	0.0838	0.957
		CDSSTR	0.1509	0.1306	0.0482	0.2191	0.0864	-0.037	0.0936	0.2677	0.1016	0.9577
		CONTIN/LL	0.1056	0.173	0.0308	0.4261	0.0809	-0.145	0.0579	0.3976	0.0742	0.9663
0.7	8	NN-LSQ	0.2755	0.1155	0.1265	0.3087	0.0348	0.1753	0.1516	0.0521	0.1704	0.8552
		SELCON3	0.1138	-0.2914	0.046	0.2832	0.0531	-0.1667	0.0866	-0.4144	0.0797	0.9649
		CDSSTR	0.1356	-0.3952	0.0506	0.4432	0.0743	-0.6562	0.0718	-0.1813	0.0889	0.9664
		CONTIN/LL	0.1067	-0.4052	0.0361	0.6878	0.0673	-0.2457	0.0688	-0.1383	0.0741	0.9685

Table S10 Performance indices for varying subsets of turn content indicated by a minimum structure content cutoff value. The number of proteins satisfying the cutoff criteria is given by column N. Cells highlighted in red indicate CDPro algorithms are the highest performing methods and cells highlighted in green indicate NN-LSQ is the best performing method.

cut(Turn)	N	Method	σ(H)	r(H)	σ(β)	r(β)	σ(T)	r(T)	σ(U)	r(U)	σ	r
0	411	NN-LSQ	0.1223	0.6953	0.1064	0.6254	0.0623	0.3225	0.1064	0.1601	0.1018	0.6006
		SELCON3	0.1156	0.7023	0.0902	0.6455	0.0537	0.294	0.0863	0.2236	0.0892	0.7147
		CDSSTR	0.1196	0.7255	0.0855	0.7117	0.0604	0.2689	0.0963	0.2519	0.0929	0.7173
		CONTIN/LL	0.1073	0.7632	0.0881	0.698	0.0557	0.2396	0.0921	0.244	0.0878	0.7286
0.1	402	NN-LSQ	0.1232	0.6707	0.1059	0.5997	0.0611	0.3253	0.1067	0.1582	0.1019	0.5795
		SELCON3	0.1099	0.729	0.0859	0.6544	0.0528	0.2288	0.0854	0.2365	0.0859	0.7226
		CDSSTR	0.1157	0.7373	0.083	0.7097	0.0594	0.2168	0.0938	0.2802	0.0903	0.7202
		CONTIN/LL	0.1063	0.7619	0.0869	0.683	0.0523	0.2454	0.0895	0.2758	0.086	0.7271
0.2	207	NN-LSQ	0.1029	0.6945	0.1006	0.6267	0.0796	0.4874	0.1335	0.1467	0.1059	0.4636
		SELCON3	0.0927	0.6837	0.0774	0.7	0.0628	0.0373	0.1121	0.101	0.0882	0.6393
		CDSSTR	0.098	0.6848	0.0724	0.7553	0.0757	0.0866	0.1229	0.0434	0.0945	0.6235
		CONTIN/LL	0.0914	0.7171	0.0805	0.7055	0.0636	-0.0783	0.1203	0.0946	0.0913	0.6309
0.3	20	NN-LSQ	0.0866	0.6613	0.0617	0.8478	0.0912	-0.2969	0.094	0.1072	0.0844	0.736
		SELCON3	0.0829	0.7039	0.0712	0.777	0.1307	-0.7941	0.1053	-0.6129	0.1001	0.6196
		CDSSTR	0.0792	0.7744	0.0714	0.8433	0.1397	-0.1234	0.1146	0.0775	0.1049	0.6258
		CONTIN/LL	0.0819	0.6846	0.0663	0.8479	0.1366	-0.6951	0.1216	0.2223	0.1055	0.5872

Table S11 PCDDB entries for the selected CD spectra. The 411 spectra are for proteins with known PDB entries.

CD0000001000	CD0000055000	CD0001158000	CD0003670000	CD0003992009	CD0003996005	CD0004000001	CD0004003011
CD0000002000	CD0000056000	CD0001159000	CD0003671000	CD0003992010	CD0003996006	CD0004000002	CD0004003012
CD0000003000	CD0000057000	CD0001160000	CD0003672000	CD0003992011	CD0003996007	CD0004000003	CD0004003013
CD0000004000	CD0000058000	CD0001161000	CD0003675000	CD0003992012	CD0003996008	CD0004000004	CD0004004000
CD0000005000	CD0000059000	CD0001162000	CD0003675001	CD0003992013	CD0003996009	CD0004000005	CD0004004001
CD0000006000	CD0000060000	CD0001163000	CD0003675002	CD0003993000	CD0003996010	CD0004000006	CD0004004002
CD0000007000	CD0000061000	CD0001164000	CD0003675003	CD0003993001	CD0003996011	CD0004000007	CD0004004003
CD0000008000	CD0000062000	CD0001165000	CD0003675004	CD0003993002	CD0003996012	CD0004000008	CD0004004004
CD0000009000	CD0000063000	CD0001166000	CD0003675005	CD0003993003	CD0003996013	CD0004000009	CD0004004005
CD0000010000	CD0000064000	CD0001167000	CD0003675006	CD0003993004	CD0003997000	CD0004000010	CD0004004006
CD0000011000	CD0000065000	CD0001168000	CD0003675007	CD0003993005	CD0003997001	CD0004000011	CD0004004007
CD0000012000	CD0000067000	CD0001169000	CD0003675008	CD0003993006	CD0003997002	CD0004000012	CD0004004008
CD0000013000	CD0000068000	CD0001170000	CD0003675009	CD0003993007	CD0003997003	CD0004000013	CD0004004009
CD0000014000	CD0000069000	CD0001171000	CD0003675010	CD0003993008	CD0003997004	CD0004001000	CD0004004010
CD0000015000	CD0000070000	CD0001172000	CD0003675011	CD0003993009	CD0003997005	CD0004001001	CD0004004012
CD0000016000	CD0000071000	CD0001173000	CD0003675012	CD0003993010	CD0003997006	CD0004001002	CD0004004013
CD0000017000	CD0000099000	CD0001174000	CD0003675013	CD0003993011	CD0003997007	CD0004001003	CD0004005000
CD0000018000	CD0000100000	CD0001175000	CD0003690000	CD0003993012	CD0003997008	CD0004001004	CD0004005001
CD0000019000	CD0000101000	CD0001176000	CD0003889000	CD0003993013	CD0003997009	CD0004001005	CD0004005002
CD0000020000	CD0000102000	CD0001177000	CD0003890000	CD0003994000	CD0003997010	CD0004001006	CD0004005003
CD0000021000	CD0000103000	CD0001178000	CD0003891000	CD0003994001	CD0003997011	CD0004001007	CD0004005004
CD0000022000	CD0000104000	CD0001179000	CD0003892000	CD0003994002	CD0003997012	CD0004001008	CD0004005005
CD0000023000	CD0000105000	CD0001180000	CD0003893000	CD0003994003	CD0003997013	CD0004001009	CD0004005006
CD0000024000	CD0000106000	CD0001181000	CD0003894000	CD0003994004	CD0003998000	CD0004001010	CD0004005008
CD0000025000	CD0000107000	CD0001182000	CD0003896000	CD0003994005	CD0003998001	CD0004001011	CD0004005009
CD0000026000	CD0000108000	CD0001183000	CD0003897000	CD0003994006	CD0003998002	CD0004001012	CD0004005010
CD0000027000	CD0000109000	CD0001184000	CD0003898000	CD0003994007	CD0003998003	CD0004001013	CD0004005011
CD0000028000	CD0000110000	CD0001185000	CD0003900000	CD0003994008	CD0003998004	CD0004002000	CD0004005012
CD0000029000	CD0000111000	CD0001186000	CD0003930000	CD0003994009	CD0003998005	CD0004002001	CD0004005013
CD0000030000	CD0000112000	CD0001187000	CD0003991000	CD0003994010	CD0003998006	CD0004002002	CD0004006000
CD0000031000	CD0000113000	CD0001188000	CD0003991001	CD0003994011	CD0003998007	CD0004002003	CD0004006001
CD0000032000	CD0000114000	CD0001189000	CD0003991002	CD0003994012	CD0003998008	CD0004002004	CD0004006002
CD0000034000	CD0000115000	CD0001190000	CD0003991003	CD0003994013	CD0003998009	CD0004002005	CD0004006003
CD0000035000	CD0000116000	CD0001191000	CD0003991004	CD0003995000	CD0003998010	CD0004002006	CD0004006004
CD0000036000	CD0000117000	CD0001192000	CD0003991005	CD0003995001	CD0003998011	CD0004002007	CD0004006005
CD0000037000	CD0000118000	CD0001193000	CD0003991006	CD0003995002	CD0003998012	CD0004002008	CD0004006006
CD0000038000	CD0000119000	CD0001194000	CD0003991007	CD0003995003	CD0003998013	CD0004002009	CD0004006007
CD0000039000	CD0000120000	CD0001195000	CD0003991008	CD0003995004	CD0003999000	CD0004002010	CD0004006008
CD0000040000	CD0000121000	CD0001196000	CD0003991009	CD0003995005	CD0003999001	CD0004002011	CD0004006009
CD0000041000	CD0000122000	CD0001197000	CD0003991010	CD0003995006	CD0003999002	CD0004002012	CD0004006010
CD0000042000	CD0000123000	CD0001198000	CD0003991011	CD0003995007	CD0003999003	CD0004002013	CD0004006011
CD0000043000	CD0000124000	CD0001199000	CD0003991012	CD0003995008	CD0003999004	CD0004003000	CD0004006012
CD0000044000	CD0000125000	CD0001200000	CD0003991013	CD0003995009	CD0003999005	CD0004003001	CD0004006013
CD0000045000	CD0000126000	CD0001201000	CD0003992000	CD0003995010	CD0003999006	CD0004003002	CD0004244000
CD0000047000	CD0000127000	CD0001202000	CD0003992001	CD0003995011	CD0003999007	CD0004003003	CD0004676000
CD0000048000	CD0000128000	CD0001203000	CD0003992002	CD0003995012	CD0003999008	CD0004003004	CD0004677000
CD0000049000	CD0001152000	CD0001204000	CD0003992003	CD0003995013	CD0003999009	CD0004003005	CD0004678000
CD0000050000	CD0001153000	CD0001205000	CD0003992004	CD0003996000	CD0003999010	CD0004003006	
CD0000051000	CD0001154000	CD0001206000	CD0003992005	CD0003996001	CD0003999011	CD0004003007	
CD0000052000	CD0001155000	CD0001207000	CD0003992006	CD0003996002	CD0003999012	CD0004003008	
CD0000053000	CD0001156000	CD0003668000	CD0003992007	CD0003996003	CD0003999013	CD0004003009	
CD0000054000	CD0001157000	CD0003669000	CD0003992008	CD0003996004	CD0004000000	CD0004003010	

Figures:

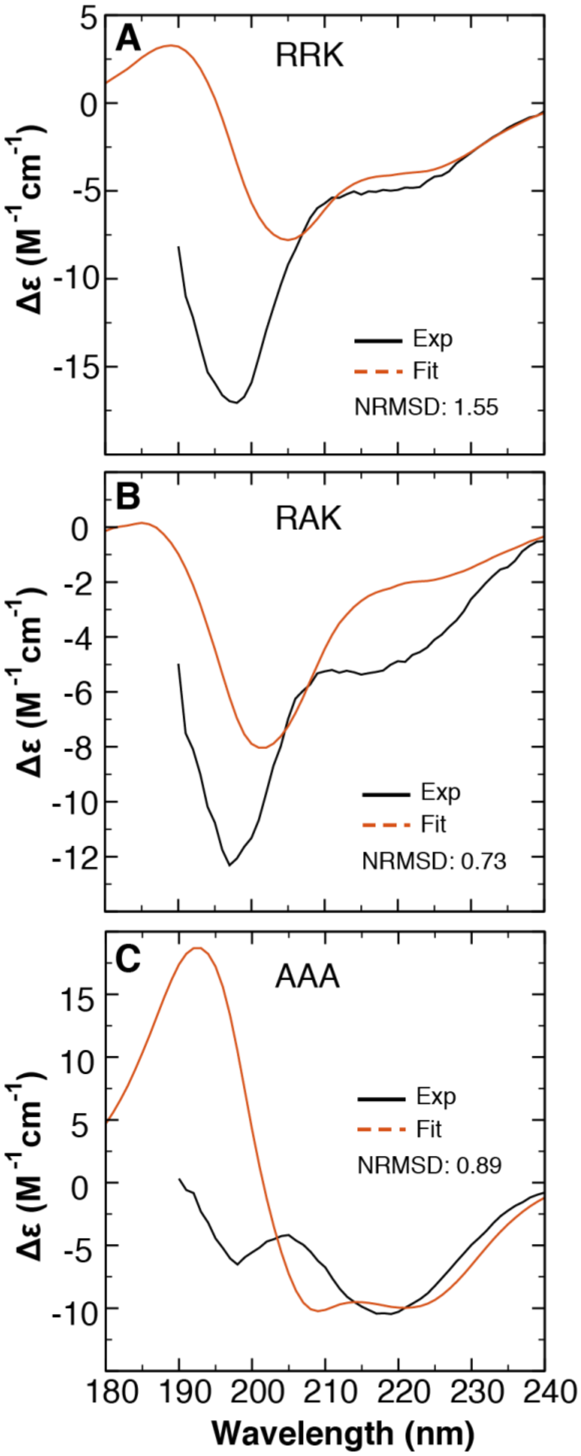

Figure S1 The calculated CD spectra derived from CAPITO for the CaMKII peptides are compared with the experimental data.

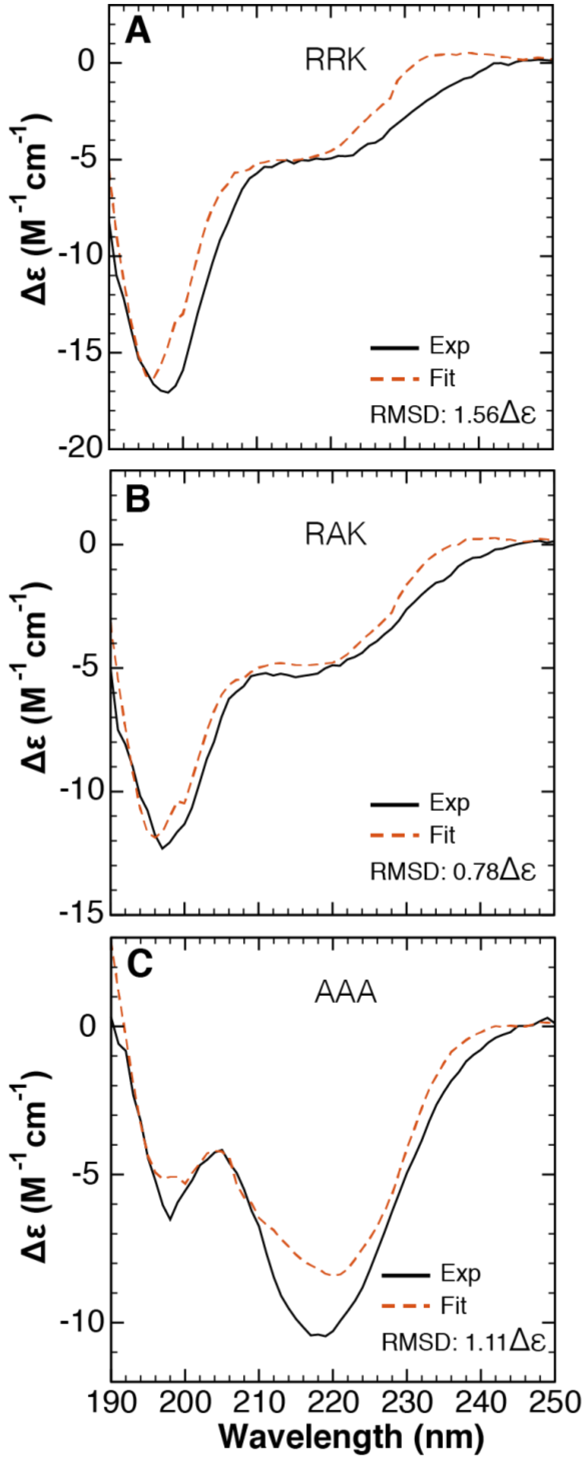

Figure S2 The predicted CD spectra derived from BeStSel for the CaMKII peptides are compared with the experimental data.

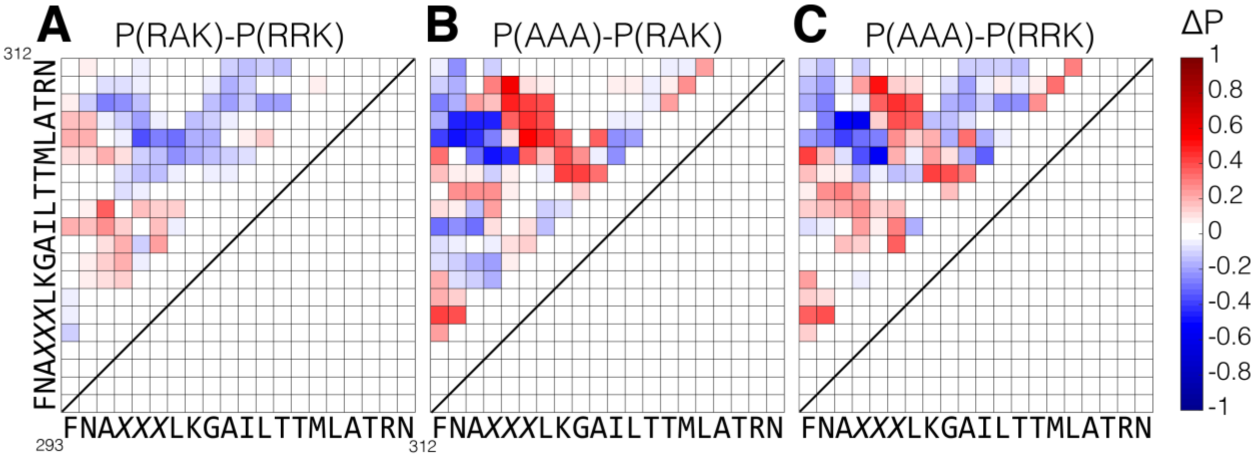

Figure S3 Difference in the probability of contact formation between the CD-refined MD structures of CaMKII peptides. Probability of contact formation are compared between peptide RAK and the wildtype RRK (a), between peptides AAA and RAK (b), and between peptide AAA and the wildtype RRK (c). The amino acid sequences are provided as the axis labels (X refers to any of the three residues RRK/RAK/AAA for corresponding peptides). The criteria of the contact formation can be found in the Method section in the main text.

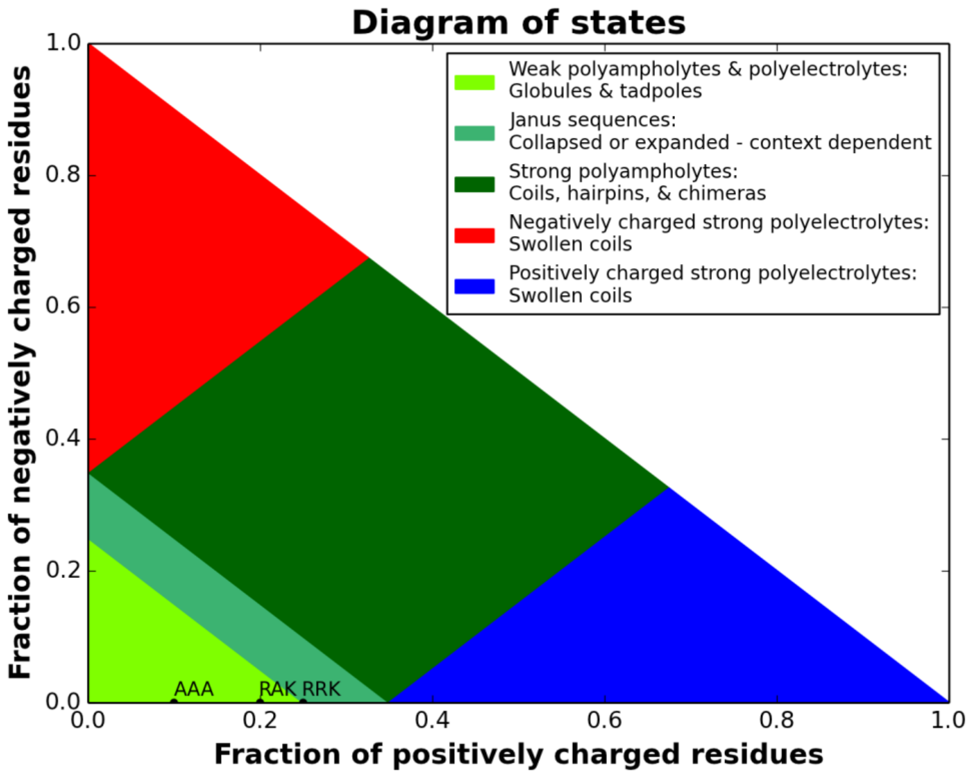

Figure S4 Diagram of states from CIDER analysis for the CaMKII peptides. The distribution of charged residues is indicative of ensemble conformation. RRK is in line with an expanded conformational ensemble, whereas RAK and AAA are predicted to more ordered.

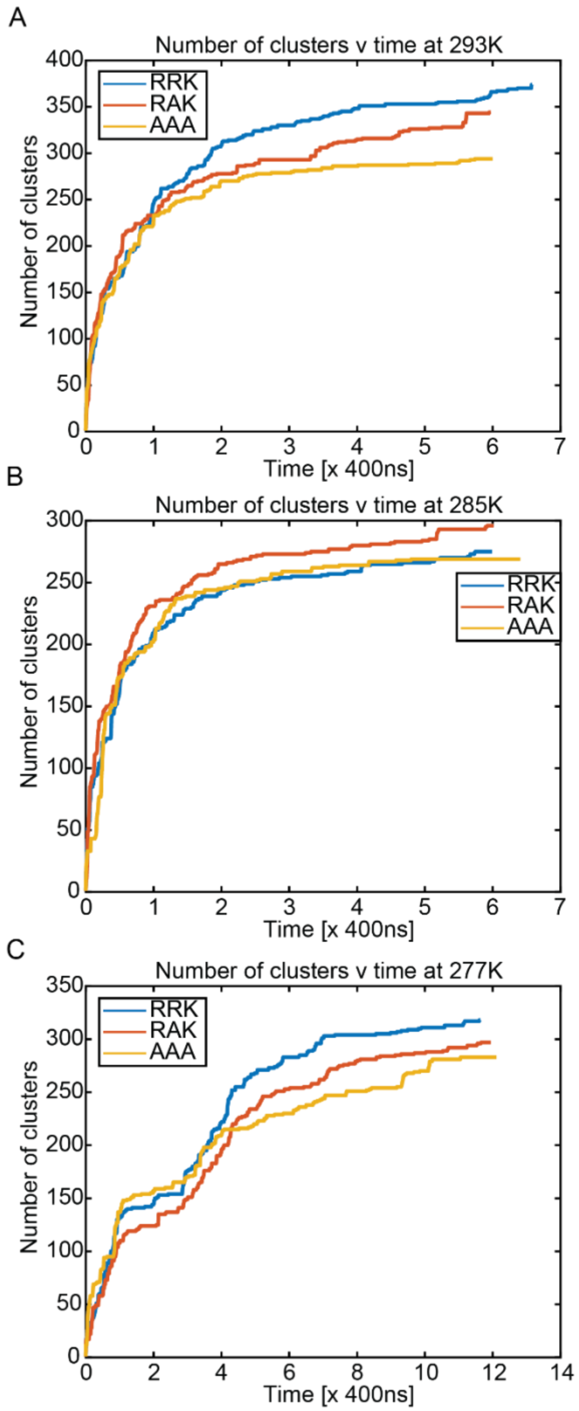

Figure S5 Clustering analysis for the MD trajectories of CaMKII peptides. Number of clusters versus accumulated time were plotted for RRK, RAK and AAA peptides at simulation temperatures of (A) 277K, (B) 285K and (C) 293K. Trajectories belonging to each peptide/temperature combination are concatenated together in chronological order and clustered using an algorithm described by Daura et al. [3]. A cutoff of 2.5 angstroms is used to distinguish clusters by root mean square distance of backbone C_α atoms. Clusters were generated using every 10_{th} frame from the MD trajectories.

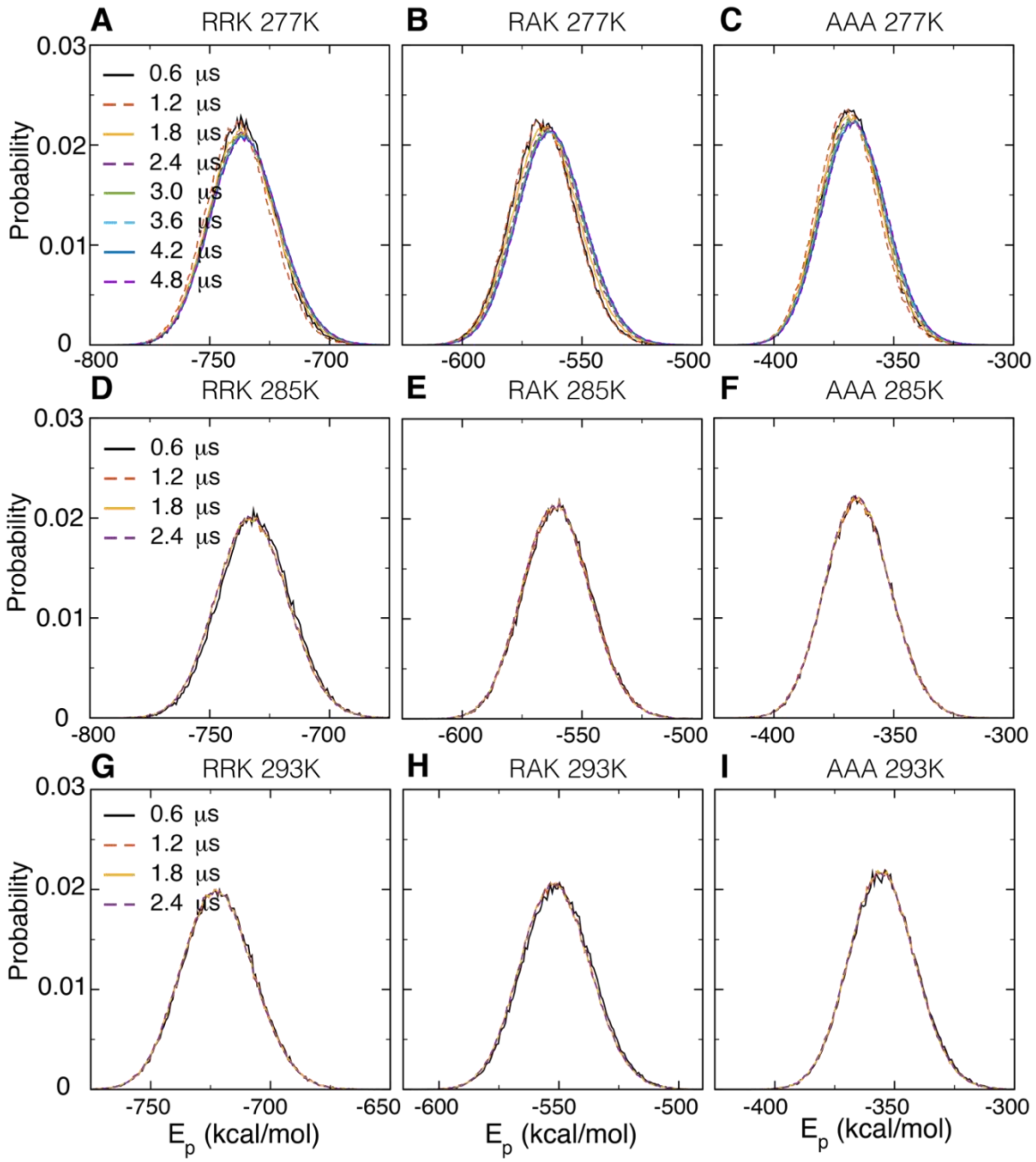

Figure S6 Probability distribution of the potential energy (E_p) for the MD trajectories of CaMKII peptides. The probability distribution of the potential energy for the peptides were compared for each peptide at temperatures of 277K (A, B, C, respectively), 285 K (D, E, F, respectively), and 293 K (G, H, I, respectively) were plotted at accumulated simulation time to show the convergence of the MD simulations.

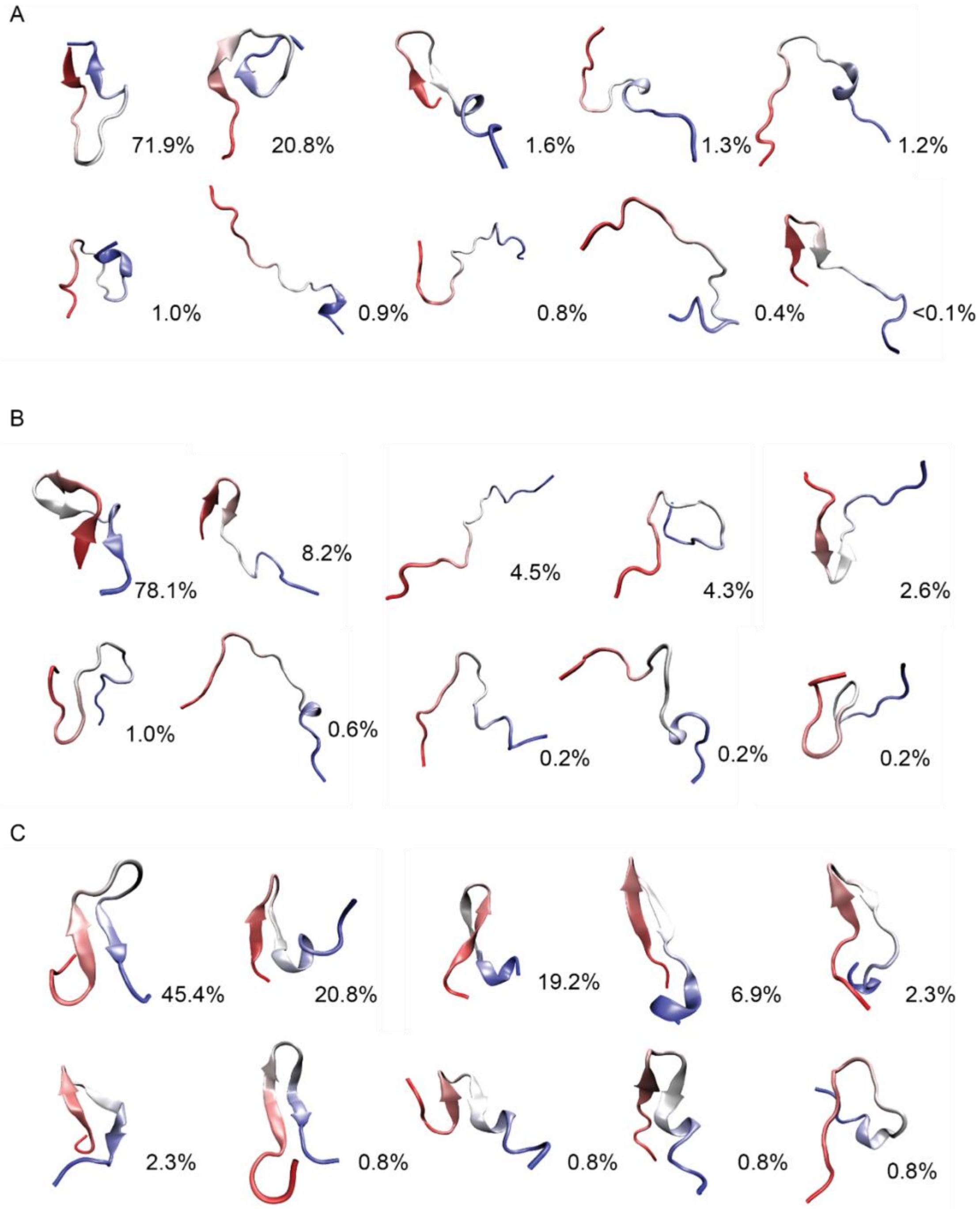

Figure S7 Sample conformations of generated ensembles using CPPTRAJ. CaMKII peptide ensembles are generated by selecting MD trajectory frames from the 293K runs with secondary structure fractions that match the NN-LSQ CD deconvolution results. 10 example structures are produced through Hieragglo clustering of the extracted ensemble using backbone Cα RMS distances. The center structures from 10 generated clusters are shown for (A) RRK, (B) RAK, and (C) AAA to illustrate the effect that each mutation has on the overall conformational behavior. The peptides are colored according to atomic index, from N-terminus (red) to C-terminus (blue), and cluster percentages are shown.

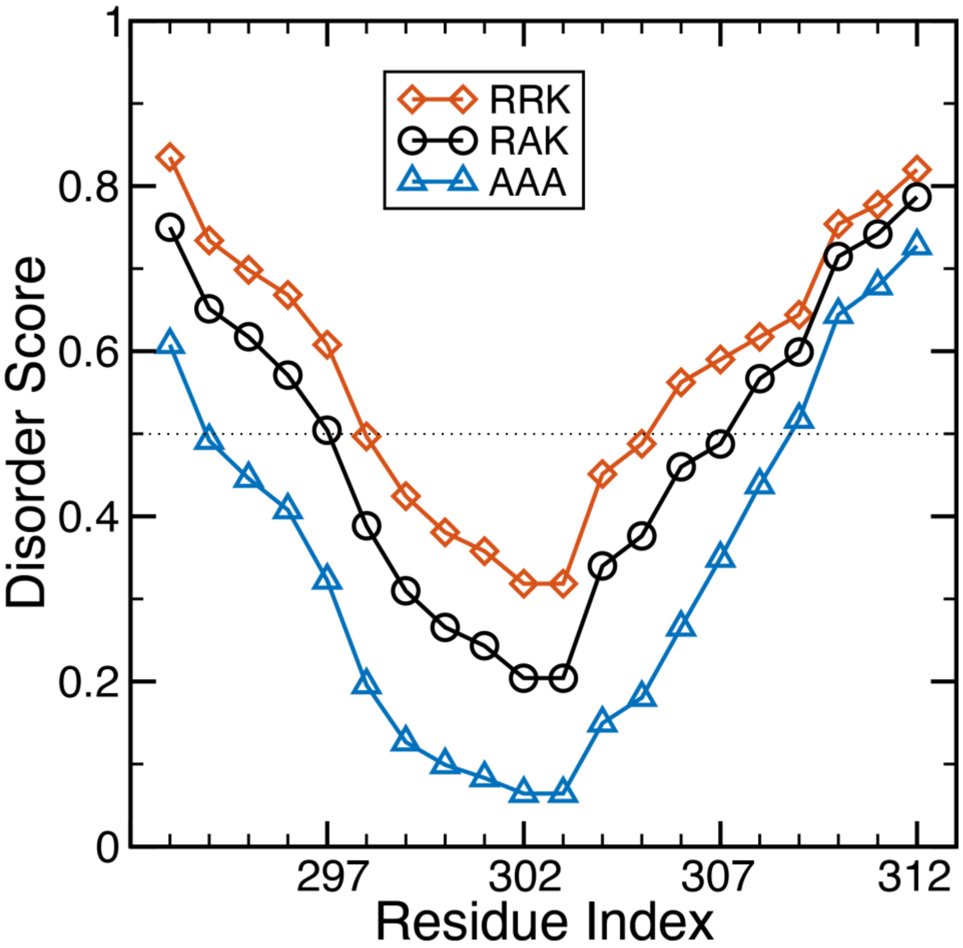

Fig. S8 Computational prediction of unstructured regions in CaMKII peptides using the IUPred web server. The predictor score is plotted against the residue number. The threshold is 0.5 and residues with a higher and lower score are considered to be in disordered and ordered regions, respectively.

Texts:

1. **Equations for CIDER analysis:**

$$\sigma = \frac{(f_{+} - f_{-})^2}{(f_{+} + f_{-})}$$

$$\sigma_i = \frac{(f_{+} - f_{-})_i^2}{(f_{+} + f_{-})_i}$$

$$\delta = \frac{\sum_{i=1}^{N} (\sigma_i - \sigma)^2}{N}$$

$$\kappa = \frac{\delta}{\delta_{max}}$$

where,
σ is the overall charge asymmetry;
f_{+}/f_{-} is the fraction of positively/negatively charged residues;
σ_i is the charge asymmetry for blob segment *i*;
δ is the squared deviation between the segmented blobs and the overall charge asymmetry;
δ_{max} is the maximum value of δ in all possible sequences for a given amino-acid composition;
N is total number of residues.

2. **Convergence analysis for the MD simulations**

Obtaining a well-sampled MD trajectory is crucial for the success of our proposed IDP ensemble generation method, therefore we ensure a well sampled conformation space through convergence analysis of the MD trajectories.

a. We use clustering analysis (Figure S5) to determine whether the majority of conformations have been sampled in our production runs. The number of clusters generated with respect to simulation time can determine the probably that the system will sample new or previously sampled conformations with additional simulation time. We observed that the change in cluster number with respect to time approaches 0 towards the end of each peptide/temperature trajectory, indicating that the majority of conformations have been sampled. A drawback to this method is that the different clustering cutoffs will change the analysis results. Larger cutoffs will not be able to distinguish minor changes in the backbone structure, resulting in small numbers of clusters and faster convergence. Similarly, smaller cutoffs are too strict and may over separate structures that should be grouped together, erroneously indicating that the trajectory diverges.
b. In addition to clustering analysis, the histograms of the distributions of the potential energy of the peptides at temperatures 277 K, 285 K, and 293 K clearly show convergence was

approached with increasing accumulated time (Figure S6). To quantically determine how the potential energy distributions change with respect to simulation time, we applied Kullback–Leibler (KL) divergence [4, 5] (Table S5). KL divergence between the probability distribution P (reference) and Q is defined as follows,

$$KL(P,Q) = -\sum_x P(x) \ln \frac{Q(x)}{P(x)}$$

KL divergence analysis indicates a well-sampled trajectory if the changes between potential energy distributions at different times is small. A value of zero indicates that the two distributions are identical. We systematically calculated the KL divergence between the distributions of accumulated trajectories at a simulation time interval of 0.6 μs. The results of our analysis indicate that all trajectories have reached convergence since the KL divergence between potential energy distributions approach to a small value of 0.01 towards the end of our simulation runs.

3. Clustering extracted peptide ensembles using CATS

Due to the large number of ensemble frames extracted from our MD trajectories, it became necessary to cluster the structures so that identifying features could be possible. In a previous study [6], we developed a clustering algorithm that was designed specifically for IDPs. A requirement of the algorithm is that the trajectory dihedral angle distributions be Gaussian-like. A histogram of the φ and ψ dihedral angles of each peptide residue (40 in total) is generated using a bin size of 3.6°. To reduce noise in the distributions, we use a Gaussian weighted moving average filter to smooth the data. We then fit Gaussian curves to the distributions and input the resulting fitting data into CATS. For our analysis of RRK, RAK and AAA, we used an ε value of 3, and 4-coordinate relaxation for all initial clusters with populations under 10% of the total ensemble size. We chose to display the top 10 clusters for each peptide ensemble, which varied with respect to accumulative size with RRK having the lowest total population and AAA having the highest total population in 10 clusters.

4. Validation of NN-LSQ deconvolution with SDP48 data set

The performance of NN-LSQ, CONTIN/LL, SELCON3, and CDSSTR deconvolution methods using SDP48 data set was evaluated using RMSD (δ) and correlation (r) coefficients defined by Woody and Sreerama [7, 8] based on 411 protein CD spectra obtained from the Protein Circular Dichroism Data Base (PCDDB) [9] with known Protein Data Bank (PDB) entries. To determine the effect of secondary structure content on the performance of each deconvolution method, the performance coefficients are calculated

for subsets of proteins with varying amounts of helix, strand, turn and unordered structure content.

Supplementary References

1. Wiedemann, C., P. Bellstedt, and M. Gorlach, *CAPITO--a web server-based analysis and plotting tool for circular dichroism data.* Bioinformatics, 2013. **29**(14): p. 1750-7.
2. Williams, R.M., et al., *The protein non-folding problem: amino acid determinants of intrinsic order and disorder*. Pac Symp Biocomput, 2001: p. 89-100.
3. Daura, X., et al., *Peptide folding: When simulation meets experiment.* Angewandte Chemie-International Edition, 1999. **38**(1-2): p. 236-240.
4. Kullback, S. and R.A. Leibler, *On Information and Sufficiency.* Ann. Math. Statist., 1951. **22**(1): p. 79-86.
5. Eguchi, S. and J. Copas, *Interpreting Kullback–Leibler divergence with the Neyman–Pearson lemma.* Journal of Multivariate Analysis, 2006. **97**(9): p. 2034-2040.
6. Ezerski, J.C. and M.S. Cheung, *CATS: A Tool for Clustering the Ensemble of Intrinsically Disordered Peptides on a Flat Energy Landscape.* J Phys Chem B, 2018. **122**(49): p. 11807-11816.
7. Sreerama, N. and R.W. Woody, *Estimation of protein secondary structure from circular dichroism spectra: comparison of CONTIN, SELCON, and CDSSTR methods with an expanded reference set.* Anal Biochem, 2000. **287**(2): p. 252-60.
8. Sreerama, N., S.Y. Venyaminov, and R.W. Woody, *Estimation of protein secondary structure from circular dichroism spectra: inclusion of denatured proteins with native proteins in the analysis.* Anal Biochem, 2000. **287**(2): p. 243-51.
9. Whitmore, L., et al., *PCDDB: the Protein Circular Dichroism Data Bank, a repository for circular dichroism spectral and metadata.* Nucleic Acids Res, 2011. **39**(Database issue): p. D480-6.
10. Dosztanyi, Z., et al., *IUPred: web server for the prediction of intrinsically unstructured regions of proteins based on estimated energy content.* Bioinformatics, 2005. **21**(16): p. 3433-4.
